# Phenomic and Transcriptomic Profiling of ZnS QD Response in *Saccharomyces cerevisiae*: A Quantum Model Organism for a Quantum Dot Study

**DOI:** 10.3390/nano16120720

**Published:** 2026-06-10

**Authors:** Sophia Luche, Luca Pagano, Marta Marmiroli, Nelson Marmiroli

**Affiliations:** 1 Department of Chemistry, Life Sciences and Environmental Sustainability, University of Parma, Parco Area delle Scienze 33/A, 43124 Parma, Italymarta.marmiroli@unipr.it (M.M.); 2 Consorzio Interuniversitario Nazionale per le Scienze Ambientali (CINSA), University of Parma, Parco Area delle Scienze 11/A, 43124 Parma, Italy; luca.pagano@unipr.it

**Keywords:** engineered nanomaterials, quantum dots, *Saccharomyces cerevisiae*, deletion mutant collection, RNA sequencing

## Abstract

Quantum dots such as CdS QDs have been extensively studied using human cells, plants, and unicellular eukaryotes such as *Saccharomyces cerevisiae*, whereas ZnS QDs—considered low-toxicity alternatives to cadmium-based nanomaterials—remain comparatively underexplored. Following preliminary analyses of ZnS QDs’ effects on wild-type *S. cerevisiae* BY4742 growth, the Yeast Knock-Out collection, comprising ~4600 haploid mutants deleted in non-essential genes, was screened in the presence of ZnS QDs. Sensitive mutants were predominantly associated with mitochondrial functions, prompting further characterization of *sod1Δ*, *glr1Δ*, and of the hypersensitive mutant *pos5Δ*. This last mutant, which lacks a mitochondrial NADH kinase, showed hypersensitivity specific to ZnS QDs but not to CdS QDs or zinc sulfate (ZnSO_4_). Flow cytometry analysis of the wild-type strain and the *pos5Δ* mutant detected no significant increase in reactive oxygen species after ZnS QD treatment. RNA-sequencing analyses of the wild-type strain and the *pos5Δ* mutant exposed to ZnS QDs (or ZnSO_4_) revealed that ZnS QD exposure selectively modulated genes encoding mitochondrial proteins, metal-binding factors, and intracellular trafficking components. Comparison with published data on CdS QDs identified specific mechanisms involving protein synthesis and degradation. *Saccharomyces cerevisiae* once again proved its versatility for studying engineered nanomaterial interactions with biological systems.

## 1. Introduction

*Saccharomyces cerevisiae* belongs to the division of Ascomycetes yeast, one of the large divisions of Fungi. Yeasts, monocellular eukaryotes with single-cell or mycelial organization, certainly represent one of the first and more important “quantum jumps” in the scale of evolution from primitive prokaryotes (bacteria). Neither animals nor plants, yeasts have a nucleus with a nuclear membrane and mitochondria like animal cells, and like plant cells, they do have vacuoles and a cell wall constituted of glucans, chitin, and proteins [1], but do not have plastids like plant cells. The endosymbiotic theory imagines the rise of yeasts as the result of a symbiotic event between different types of bacteria. The one that is included inside the other one (*endo*) evolved specialized functions (respiration, oxidative phosphorylation), losing part of its genome while maintaining few genetic functions into what is now called the mitochondrial DNA (mtDNA) [2].

The use of Alternative Testing Strategy (ATS) and screening systems in toxicological studies has been represented through the 3Rs principle, which aims to replace, reduce, and refine animal testing [3]. In vitro tools such as monocellular or pluricellular eukaryotic model systems present the advantage of providing a physiological assessment and a potential correlation with the genetic base of the phenomenon, with a high level of robustness in terms of results quantitation, reliability, and scalability to higher eukaryotes, reducing costs to satisfy public opinion. Among the most relevant model organisms, *Saccharomyces cerevisiae* (yeast), *Caenorhabditis elegans* (nematode), and *Mus musculus* (mouse) have been utilized as functional tools for the understanding of metabolic pathways, human diseases (particularly those of mitochondrial origin), and nervous system organization [4].

In particular, the relevance of yeast as a model organism is paramount: its genome, proteome, and metabolome have been fully characterized; its mtDNA and mitochondrial functions, as well as cell cycle dynamics, are well understood, and its genetics are facilitated by the largest collections of single-gene deletion mutants [5]. Due to its ease of genetic manipulation, yeast has proved to be an optimal recipient for many genes involved in or correlated with important human diseases [6,7]. Moreover, the advantages of yeast as a quantitative model in environmental toxicology are its short life cycle and rapid reproduction, which allow testing on large clone populations under any conditions: water, soil, food, materials [8].

Since engineered nanomaterials (ENMs), including quantum dots (QDs), have been linked to the development of cell disorders and disease, *S. cerevisiae* has been exploited as a platform for nanotoxicity studies, and its ability to internalize ENMs through endocytic processes has enabled the dissection of different steps in exposure pathways and response mechanisms in such a rapidly evolving field: from the morpho-functional point of view, it is important to highlight how the cell wall does not affect the dynamics of QD internalization since the mechanisms employed for the uptake are generally regulated by vesicle trafficking and from QD physico-chemical stability [8,9,10,11], even if permeability of the membrane can be, for specific experimental necessities, artificially increased through spheroplast or protoplast formation (which may regenerate their cell wall) or antibiotics administration (e.g., nystatin, able to create pores in the cell wall) [8,10]. Another consideration can be related to the formation of buds from the mother cell, which creates temporary areas of cell wall breakage followed by cell wall regeneration [5]. The use of the Yeast Knock-Out (YKO) collection (mutants that show deletions in single functions) for screening purposes has the main advantage of characterizing tolerance and sensitivity phenomena related to specific genes, pathways, and phenotypes [5,10]. The dualism genotype/phenotype becomes the paradigm of modern toxicology, in which taxonomical description of the phenomena at the morphological and physiological levels is no longer sufficient, and where the primary objective becomes the research of molecular targets [3,4,5], with the objective of testing materials that are safer by design, developing ligands for intracellular targets, and producing more sustainable molecules or materials that are able to be degraded in the environment [3,4].

Zinc sulfide quantum dots (ZnS QDs) are often used as the shell layer in core-shell structures because they enhance quantum yield, protect the core from oxidation, and improve stability [12,13]. ZnS QDs are considered less toxic and more biocompatible than the metals that usually constitute the core, primarily because the shell prevents toxic metal ions from being released from the core [14,15], such as with CdS QDs, which induce cytotoxicity in plant and cellular models [16,17,18]. Few studies have been reported in the literature that investigate the effects of ZnS QDs *per se*. In HeLa cell lines, ZnS QDs were shown to decrease cell viability with increasing concentration, while maintaining cell survival rates above 90% at a concentration of 100 mg L^−1^ [19]. In plants, it has been observed that the treatment of two metal hyperaccumulating plant species, *Noccaea caerulescens* L. and *Thlaspi perfoliatum* L., with ZnS QDs does not harm plant growth and leaf morphology [20], nor does it damage tomato (*Solanum lycopersicum* L.) plants [21]. From a study in *Danio rerio*, in which core-only QDs composed of ZnS or CdS were tested alone and in combination, it emerged that CdS QDs caused markedly more oxidative stress than ZnS QDs, and that both manifested bioaccumulation in tissues following an exposure of 7 days [22]. Taking into account the physico-chemical properties of quantum dots, which largely differ from other metal and metal oxide nanoparticles in terms of size, shape, charge, and stability, the response to ZnS QDs has been compared with that to ZnSO_4_, to mimic the complete availability of Zn ions (to understand if the effects can be related to Zn release) and to compare the results to other types of QDs that have been previously studied with the same systems biology approach [10,23,24]. The present study combined different -omics approaches, YKO collection screening, and transcriptional profiling to elucidate the cellular response to ZnS QDs and to compare with CdS QDs using *Saccharomyces cerevisiae* as a model organism [10,23,24]. The rationale for the use of the deletion mutant collection for cellular and molecular studies is illustrated in Figure S1. The relevant literature on yeast as a model system for nanotoxicology purposes is reported in Table S1.

## 2. Materials and Methods

### 2.1. Nanomaterial Synthesis and Characterization

ZnS QDs and CdS QDs were manufactured by IMEM-CNR (Parma, Italy) and were previously characterized (Table S2) through dynamic light scattering (DLS), ζ-potential, and dissolution. Detailed synthesis and characterization have been reported in Pagano et al. (2022) [18]. Summarizing, for ZnS QDs, anhydrous ZnCl_2_ (10 mmol, Merck, Darmstadt, Germany) is dissolved into 200 mL of ethylene glycol (EG) and heated to 160 °C (S1) in a three-neck flask under Ar atmosphere. In another flask, purged with Ar, 10 mmol of thiourea is dissolved into another 50 mL of EG (S2) at 80 °C. Under vigorous magnetic stirring, S2 is then quickly injected into S1. The mixed solution suddenly turns from clear to milky white, indicating the formation of ZnS nanocrystals. The solution is allowed to rest at 150 °C for 10 min and then brought to room temperature (RT) using an ice bath. The ZnS nanocrystals are separated from the reaction solution by centrifugation, washed with acetone and ethanol, and finally dried in a desiccator. Powder X-ray diffraction data were collected on a Rigaku Smartlab XE diffractometer (Rigaku, Tokyo, Japan) equipped with an HyPix3000 detector operating in 1D mode by using Cu Kα = 1.5046 Å wavelength [18]. The Cu Kβ line was removed using a Ni filter and, for the iron-bearing samples, XRF reduction mode was employed. Data collection was carried out in Bragg–Brentano geometry in the 2θ x-y° range, step size 0.1°. Mean QD dimensions were estimated by the Sherrer formula, using a weight-average mean of reflection intensity. Further details are reported in Pagano et al. (2022) [18]. The average particle size (dh) and zeta (ζ) potential of the ENMs (100 mg L^−1^) were determined in ddH_2_O on a Zetasizer Nano Series ZS90 (Malvern Instruments, Malvern, UK). ZnS QD dissolution was investigated by ultracentrifugation of a standard solution (100 mg L^−1^) prepared in ddH_2_O maintained for 20 days. Ultracentrifugation was performed at 30,000 rpm for 10 min at 20 °C (Optima Max-XP Ultracentrifuge, Beckman-Coulter Inc., Brea, CA, USA). Aliquots (1 mL) of the solutions obtained were digested in 4 mL of 1 M HNO_3_ for 20 min at 200 °C using a VELP DK20 digester (VELP Scientifica, Usmate, Italy). The digested solutions were analyzed for metal content through flame atomic absorption spectroscopy (FA-AAS; AA240FS, Agilent Technologies, Santa Clara, CA, USA). More details are reported in Pagano et al. (2022) [18]: mean size: 3–5 nm; zeta (ζ) potential: +12.0; average particle size (dh) in ddH_2_O: 302.9 nm; purity (%): 99.99; Metal (%): 63.2; dissolution rate (%): 1–3. ZnS QDs were analyzed by transmission electron microscopy (TEM, Talos F200S G2, SEM FEG Thermo Fisher Scientific, Waltham, MA, USA), as reported in Figure S2. Before use, the ENMs were suspended in sterile milliQ water to obtain a 2.5 mg mL^−1^ stock solution and sonicated for 15 min at room temperature in an Elma Transsonic T460/H (Elma Electronic GmbH, Pforzheim, Germany).

### 2.2. Yeast Strain and Growth Conditions

The yeast *S. cerevisiae* wild-type strain used in this study is BY4742 (*MATα his3Δ1 leu2Δ0 lys2Δ0 ura3Δ0*). The Yeast Knock Out (YKO) collection is derived from the *Saccharomyces* Genome Deletion Projects and consists of 4667 haploid *MATα* single-gene deletion mutants constructed by the kanMX disruption-deletion cassette, stored in 96-well plates. The YKO collection deletion mutants whose phenotypes have been analyzed by serial dilution spot assays are *pos5Δ*, *sod1Δ*, and *glr1Δ*. Growth media included YPD (1% *w*/*v* yeast extract, 2% *w*/*v* peptone, 2% *w*/*v* dextrose), YPG (1% *w*/*v* yeast extract, 2% *w*/*v* peptone, 2% *w*/*v* glycerol), YPD-agar (2% *w*/*v* agar) and YPG-agar. To estimate the sub-inhibitory concentration to be utilized in YKO screening (100 mg L^−1^), a preliminary assessment of the ZnS QDs inhibitory concentration was performed in the ranges of 0–200 mg L^−1^ in liquid culture media and 0–1000 mg L^−1^ on solid culture media. Validation of these concentrations is reported in the serial dilution spot assay and 96-well plate growth assay sections.

#### 2.2.1. Screening of the YKO Collection

After preliminary analyses on wild-type growth, which identified 100 mg L^−1^ ZnS QDs as the highest tested concentration that did not significantly inhibit wild-type growth, the screening of the YKO collection was performed in YPD medium supplemented with 100 mg L^−1^ of ZnS QDs or sterile milliQ water (untreated control). Each well contained a final volume of 200 µL, consisting of 192 µL of YPD medium and 8 µL of either QD stock suspension or sterile milliQ water. Starting from pre-culture copies of the YKO master plate, single mutants were inoculated in 96-well plates with the help of a sterile 96-pin replicator (V & P Scientific, Inc., San Diego, CA, USA). After a 48 h incubation at 28 °C, growth was quantified spectrophotometrically by measuring optical density at 600 nm (OD_600_) with an iMark™ Microplate Absorbance Reader (Bio-Rad, Hercules, CA, USA), and a growth index was calculated for each mutant. Mutants were classified as sensitive if they showed a 30% growth reduction (Student’s *t*-test, *p* < 0.05) compared to the control, not including slow-growth mutants and dubious open reading frames. Gene Ontology and Functional Classification analyses (Tables S3 and S4) of genes deleted in ZnS QD-sensitive mutants were performed with YeastMine and GO Slim Mapper tool (*Saccharomyces* Genome Database, https://www.yeastgenome.org/ accessed on 29 April 2026). Chord diagrams were created with R (version 2025.05.0, build 496) *circlize* package [25].

#### 2.2.2. Serial Dilution Spot Assay

For the serial dilution spot assay, yeast strains were pre-cultured in YPD to log-phase at 28 °C, with constant shaking. Cells were diluted to OD_600_ values of 1 and 3 µL of serial dilutions in 10-fold increments (10^7^–10^3^ cells mL^−1^) and were spotted into YPD-agar, SD-agar, and YPG-agar Petri dishes supplemented with ZnS QDs (100, 200, 500 or 1000 mg L^−1^), ZnSO_4_ (294 mg L^−1^), CdS QDs (25, 100 or 200 mg L^−1^), or sterile milliQ water as untreated control, as specified in each condition. The ZnSO_4_ concentration was calculated, based on the ratio of the respective molecular weights, to match the zinc quantity present in the growth medium supplemented with 100 mg L^−1^ of ZnS QDs. Due to their higher toxicity, the concentration range used for CdS QDs started from a lower concentration (25 mg L^−1^). Growth was monitored and recorded after 48 h (YPD-agar) or 72 h (SD-agar, YPG-agar) of incubation at 28 °C.

#### 2.2.3. Growth Assay in 96-Well Plates

Yeast strain pre-cultures were diluted to an OD_600_ value of 0.05 and incubated in 96-well plates with YPD supplemented with increasing concentrations of ZnS QDs (10–200 mg L^−1^) or ZnSO_4_ (29–294 mg L^−1^). Growth quantification was performed by measuring OD_600_ of the cultures over 48 h, at 2 h intervals. The time point at which untreated cell cultures exited the exponential phase was selected to collect growth data for constructing histograms and dose–response curves. Statistical analysis consisted of *t*-tests performed with Past 4.12b Software. Statistical significance is indicated in the figures as follows: * *p* < 0.05, ** *p* < 0.01, *** *p* < 0.001, **** *p* < 0.0001; differences with *p* ≥ 0.05 were considered not significant (ns).

#### 2.2.4. Colony Forming Unit Quantification

To assess yeast vitality following exposure to ZnS QDs, colony forming unit (CFU) quantification was performed. Briefly, the yeast strains pre-cultured in YPD were diluted to an OD_600_ value of 0.05, transferred to 96-well plates, and incubated at 28 °C in YPD supplemented with ZnS QDs (50 mg L^−1^) or sterile milliQ water (untreated control), to test their viability using half the sub-inhibitory concentration. After 24 h, cells were collected, quantified through a Bürker counting chamber, and diluted to 10^3^ cells mL^−1^, and aliquots (50 µL) were plated in sterile YPD-Agar plates. The number of CFUs was recorded after a 48 h incubation at 28 °C.

### 2.3. Flow Cytometry

Flow cytometry analyses (Table S5) were performed to assess whether the treatment with ZnS QDs could cause cell death or induce the production of reactive oxygen species (ROS) in wild-type and *pos5Δ* mutants. Wild-type BY4742 and *pos5Δ* cells pre-grown in YPD were inoculated in YPD supplemented with ZnS QDs (100 mg L^−1^) or sterile milliQ water and incubated at 28 °C. After 24 h, aliquots of 10^7^ cells mL^−1^ were collected and incubated with 20 µM 2′7′-dichlorodihydrofluorescein diacetate (H_2_DCFH-DA) and 5 µg mL^−1^ propidium iodide (PI) for 20 min in the dark. Aliquots of cells incubated for 10 min at 95 °C or with 0.03% (*v*/*v*), 0.3% (*v*/*v*) or 3% (*v*/*v*) hydrogen peroxide (H_2_O_2_) (Merck, Darmstadt, Germany) before staining were used as positive controls for the quantification of dead cells and ROS formation, respectively. Fluorescence was detected with a NovoCyte^®^ flow cytometer (ACEA Biosciences, Inc., San Diego, CA, USA).

### 2.4. RNA Extraction, Sequencing, and Bioinformatic Analyses

For mRNA-sequencing analyses, wild-type and *pos5Δ* strains were cultured in YPD supplemented with ZnS QDs or ZnSO_4_ (26 mg L^−1^ or 75.4 mg L^−1^, respectively), and RNA was extracted at 5 and 24 h of treatment using the RNeasy Mini Kit (QIAgen, Hilden, Germany), in triplicate. Considering the *pos5Δ* hypersensitivity, a sub-lethal concentration of ZnS QDs was chosen. After quality control of the RNA samples by gel electrophoresis and by determining 260/280 ratio with a Nanodrop Lite Spectrophotometer (Thermo Fisher Scientific, Wilmington, DE, USA), the samples were sequenced (IGA Technologies Services, Udine, Italy). Sequencing was performed by IGA Tech service (Udine, Italy). Libraries were prepared using a Universal Plus mRNA-Seq kit (Tecan Genomics, Redwood City, CA, USA), and the samples were quality-tested and quantified by Agilent 2100 Bioanalyzer RNA assay (Agilent technologies, Santa Clara, CA, USA). A Qubit 2.0 Fluorometer (Invitrogen, Carlsbad, CA, USA) and Agilent Bioanalyzer DNA assay were used for final library checking, and libraries were prepared and sequenced in paired-end 150 bp mode on a NovaSeq 6000 (Illumina, San Diego, CA, USA). Base calling, demultiplexing, and adapter masking were performed with an Illumina BCL Convert v3.9.3, and trimming was performed by ERNE-BS5 software. The reads were aligned on reference *S. cerevisiae* genome GCF_000146045.2_R64 with STAR. Assembly and quantitation of full-length transcripts representing multiple spliced variants for each gene locus were performed with Stringtie. The RSeqQC package was used for quality control (Figure S3). Preliminary statistical analyses on dataset distribution, normalization, PCA, box plots, and MA-plots were performed with R statistical software (www.r-project.org accessed on 29 April 2026) and are reported in Figures S4–S6, respectively. Thresholds for differentially expressed genes were established at 1.5 fold-change and FPKM (Fragments Per Kilobase Million) higher than 50 (Figure S7). Pairwise comparisons were performed for both the 5 h (early response) and the 24 h (late response) treatments: the wild-type strain exposed to ZnS QDs or ZnSO_4_ was compared against the untreated wild-type strain, and likewise, the *pos5Δ* mutant exposed to ZnS QDs or ZnSO_4_ was compared against the untreated *pos5Δ* mutant. In addition, the untreated *pos5Δ* mutant was compared with the untreated wild-type strain. Venn diagrams (Figure S8) were generated to compare genes modulated in different tested conditions, with Venny Bioinformatics tool (https://bioinfogp.cnb.csic.es/tools/venny/ accessed on 29 April 2026). Up-regulated and down-regulated genes identified in different conditions were analyzed by the Functional Annotation Chart (Table S6) to identify enriched annotation terms (*p* < 0.05; Benjamini < 0.05) using the DAVID Bioinformatics Database (https://davidbioinformatics.nih.gov accessed on 29 April 2026). DAVID default annotation categories were condensed into 3 groups: Biological Process (UP_KW_BIOLOGICAL_PROCESS, GOTERM_BP_DIRECT), Cellular Component (UP_KW_CELLULAR_COMPONENT, GOTERM_CC_DIRECT), and Molecular Function (UP_KW_MOLECULAR_FUNCTION, GOTERM_MF_DIRECT). Functional Annotation Chart bubble plots and heatmaps were generated with R Studio (version 2024.12.1). Network analyses were performed using the GeneMANIA Cytoscape plugin (version 3.5.3).

## 3. Results and Discussion

### 3.1. Exposure of Wild-Type Strain to ZnS QDs

**Figure 1:**
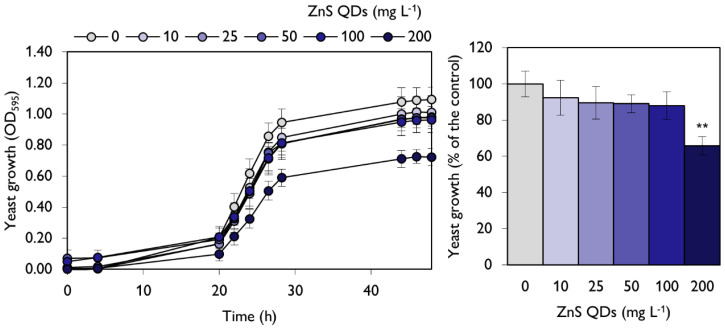
ZnS QDs inhibit wild-type *S. cerevisiae* growth at high concentrations. Growth curves (***left***) of wild-type BY4742 grown in YPD medium supplemented with increasing concentrations (10–200 mg L^−1^) of ZnS QDs. Histogram (***right***) with comparison of percentage growth after 48 h across conditions (Student’s *t*-test, ** *p* < 0.01; Past 4.12b).

To investigate the impact of ZnS QDs on *S. cerevisiae* wild-type growth, a 96-well-based growth assay in liquid culture conditions was performed. Wild-type growth in YPD supplemented with increasing concentrations of ZnS QDs (0–200 mg L^−1^) was recorded for 48 h by measuring the culture’s optical density at 595 nm (OD_595_) to obtain growth curves for each tested condition (Figure 1). The analysis showed a plateau reduction in the wild-type strain, with growth inhibition of greater than 30% following exposure to 200 mg L^−1^ ZnS QDs.

These preliminary analyses in the wild-type strain revealed a degree of growth inhibition that supported the general belief that ZnS QDs were less toxic, as compared to other metal-based forms of QDs, such as CdS QDs, which, when tested in similar conditions in this model organism, showed significantly lower minimal inhibitory concentrations, both in solid and liquid growth media [26,27]. Serial dilution spot assay analyses of the haploid wild-type strain exposed to CdS QDs revealed a strong inhibition already at 20 mg L^−1^ [26]. In liquid media, 24 h treatment of diploid wild-type *S. cerevisiae* with 10 mg L^−1^ CdS QDs reduced yeast growth by more than 50% [27].

### 3.2. Screening of the YKO Collection

**Figure 2:**
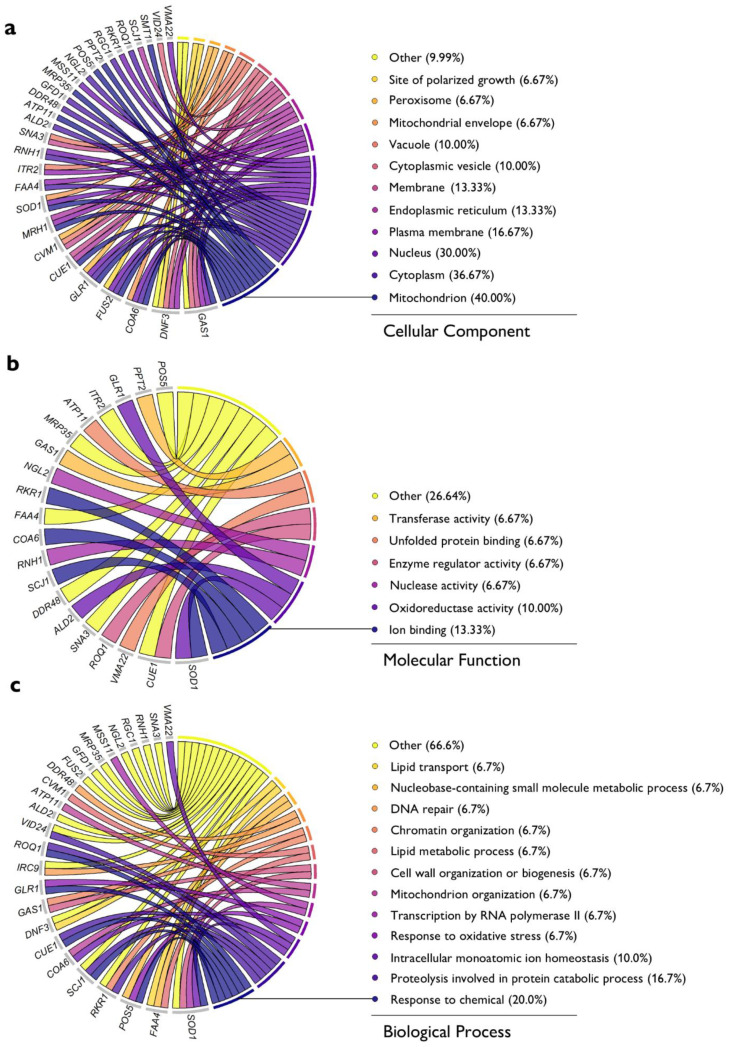
Gene Ontology classification of genes deleted in ZnS QD-sensitive mutants. Chord diagrams illustrate cellular components (**a**), molecular functions (**b**), and biological processes (**c**), generated using the Saccharomyces Genome Database (SGD) Slim Term Mapper. GO terms associated with only one gene are grouped as “other”.

YKO collections of *S. cerevisiae* have served as a platform to study ENM effects in vivo, including CdS QDs, CuO NPs, Au NPs, Ag NPs, and ZnO NPs [9,10,11,28,29]. The YKO collection used in this study consisted of a set of ~4600 haploid mutants deleted in non-essential genes, stored in 96-well plates. The screening was performed in YPD supplemented with 100 mg L^−1^ ZnS QDs, and growth was quantified spectrophotometrically (OD_595_) with a Microplate Reader. Gene Ontology analysis of genes deleted in the 30 sensitive mutants (Table S3) revealed mitochondrial compartments (40%), cytoplasm (36.67%), and nucleus (30%) as the most-affected cellular compartments (Figure 2a). Key biological processes included chemical stress response (20%), oxidative stress response (6.67%), protein catabolism (16.67%), and ion homeostasis (10%), while molecular functions involved ion binding (13.3%) and oxidoreductase activity (10%) (Figure 2b,c; Table S4).

These findings align with prior CdS QD studies. Using the same YKO collection, Marmiroli et al. (2016) identified oxidative stress and mitochondrial organization as critical in the CdS QD response [10]. Pasquali et al. (2017) showed CdS QDs induce mitochondrial disruption, reduce GSH:GSSG ratios, and impair electron transport chains [23]. They also identified nuclear genes that play an important role in mitochondrial organization, including *TOM5*, which is overexpressed after exposure to CdS QDs, and whose deletion results in increased sensitivity to CdS QDs [23]. In this study, a set of genes (*ATP11*, *COA6*, *CUE1*, *FUS2*, *GAS1*, *GLR1*, *MRH1*, *MRP35*, *POS5*, *PPT2*, *SMT1*, *SOD1)* encoding mitochondrial proteins was identified, including some specifically linked to oxidative stress response (*GLR1*, *SOD1*), underscoring how mitochondrial functions can be involved in detoxification mechanisms induced by exposure to different types of quantum dots.

### 3.3. Phenotypic Analyses of glr1Δ, sod1Δ, and pos5Δ

#### 3.3.1. Growth of *pos5Δ*, *sod1Δ*, and *glr1Δ* Exposed to ZnS QDs

Several studies on the effects of metal-based QDs highlight their ability to induce oxidative stress in different model organisms, as in the case of CdSe QDs, CdTe QDs, ZnO QDs, and CdS QDs [16,30,31,32]. In addition, many studies identify mitochondria as one of the main targets of quantum dot toxicity in plant, unicellular, and animal models [18,23,33,34,35]. A similar study by Ayer et al. (2012) [36], in which a YKO collection was screened with the use of a GFP-based redox probe capable of targeting specific organelles, highlights how some yeast genes are crucial for the maintenance of the redox potential in different organelles. Specifically, the authors identify glutathione reductase Glr1, the superoxide dismutase Sod1, and the mitochondrial NADH-kinase Pos5 as key factors in subcellular redox homeostasis. Pos5 is exclusively localized in mitochondria, whereas Glr1 and Sod1 are also involved in cytosolic and peroxisomal redox balance [36]. As these three genes play important roles in the detoxification from reactive oxygen species and the maintenance of redox homeostasis in cytosol, peroxisome, and mitochondria, *sod1Δ*, *glr1Δ*, and *pos5Δ* were selected to further dissect their roles in response to ZnS QDs.

**Figure 3:**
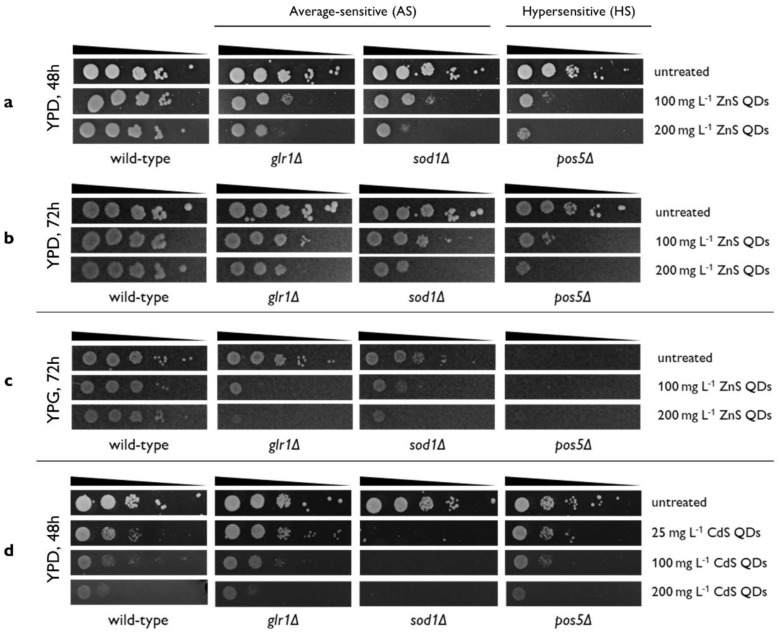
QD effects on different carbon sources. Serial dilution spot assay (10^7^–10^3^ cells mL^−1^) of yeast grown on YP agar containing 2% dextrose (YPD) or 2% glycerol (YPG) as carbon source, supplemented with ZnS QDs (100–200 mg L^−1^), CdS QDs (25–200 mg L^−1^), or sterile Milli-Q (control). Growth was recorded after 48 h (**a**,**d**) or 72 h (**b**,**c**) to ensure full colony development.

Serial dilution spot assays were used to further confirm their sensitivity to ZnS QDs, and quantification of colony forming units (CFU) reflected their different degrees of sensitivity (Figures S9 and Figure 3a,b). While *sod1Δ* and *glr1Δ* were classified with average sensitivity (AS, with no or low growth at 10^4^ cell mL^−1^), *pos5Δ* was attributed a phenotype of hypersensitivity (HS, with no or low growth at 10^7^ cell mL^−1^). In addition to their defense roles against oxidative stress, proteins encoded by *SOD1*, *GLR1*, and *POS5* are also involved in other biological processes that could contribute to the sensitivity to ZnS QDs exhibited by these deletion mutants. For example, Sod1 participates in the cellular homeostasis of zinc ions, the regulation of respiration processes, and the organization of the cell wall [37,38,39]. Glr1 is directly involved in GSH generation, and the latter is important in the maintenance of mitochondrial genome integrity and in growth and sporulation processes [40]. Moreover, GSH acts as a chelating agent in vacuolar metal sequestration processes, and could be involved in zinc, nickel, and chromium uptake by *S. cerevisiae* [41,42]. Pos5, besides providing mitochondria with the reducing power of NADPH, is required for iron-sulfur cluster biogenesis and mitochondrial DNA stability [43,44].

#### 3.3.2. Involvement of Mitochondrial Functions in *pos5Δ*, *sod1Δ*, and *glr1Δ* Sensitivity to QDs

To assess mitochondrial involvement in ZnS QD sensitivity, *glr1Δ*, *sod1Δ*, and *pos5Δ* were grown and tested in glycerol, a non-fermentable but respirable carbon source (Figure 3c). Wild-type growth slightly decreased when exposed to ZnS QDs in the presence of glycerol as sole carbon source. As reported in a previous study, CdS QDs inhibit wild-type growth in glycerol. While mitochondrial functions responded to both QD types, CdS QDs additionally disrupted the respiratory chain [23]. *sod1Δ* growth declined sharply in glycerol even at low CdS QD concentrations, consistent with Sod1’s role in ROS detoxification and respiratory regulation [39]. *glr1Δ* growth was less affected in glycerol, as *glr1Δ* is not respiratory-deficient. Studies suggest glutathione metabolism (*GSH1*, *GSH2*, *GLR1*) supports CdSe QD synthesis in yeast, where reduced glutathione (GSH) stabilizes QDs or reduces toxicity [45]. In glycerol, heightened mitochondrial activity, redox shifts, and low GSH availability may explain *glr1Δ*’s increased ZnS QD sensitivity. *pos5Δ* failed to grow in non-fermentable carbon sources due to mitochondrial dysfunction, underscoring *POS5*’s role in mitochondrial DNA stability [43]. Growth analysis of these mutants indicates that the varied sensitivity phenotypes to ZnS QDs in glycerol media likely stem from multiple factors, all relating to each mutant’s capacity for respiratory metabolism to sustain growth.

#### 3.3.3. Sensitivity of *pos5Δ*, *sod1Δ*, and *glr1Δ* to ZnS QDs and CdS QDs

Serial dilution spot assays were used to compare the sensitivity of *glr1Δ*, *sod1Δ*, and *pos5Δ* mutants to CdS QDs and ZnS QDs (0–200 mg L^−1^; Figure 3d). The wild-type strain showed growth inhibition at 25 mg L^−1^ CdS QDs, confirming CdS QDs’ greater toxicity compared to ZnS QDs. *glr1Δ* exhibited higher tolerance to CdS QDs than the wild type, suggesting Glr1 absence may liberate cells from a key CdS QD target, enabling alternative detoxification. *sod1Δ* growth was fully inhibited by CdS QDs, indicating Sod1’s critical role in mitigating CdS QD toxicity. *pos5Δ* showed slightly greater inhibition than the wild type, implying *POS5* deletion does not significantly enhance sensitivity or tolerance to CdS QDs. Therefore, *POS5* can be considered a breaking point in quantum dot tolerance/sensitivity between CdS QDs and ZnS QDs, and it cannot be attributed to the different toxicity of the metal core because Cd is more toxic than Zn, but the absence of *POS5* makes ZnS QD sensitivity higher than CdS QD sensitivity.

### 3.4. pos5Δ Hypersensitivity

The mitochondrial NADH kinase Pos5 plays critical roles in mitochondrial DNA stability, iron-sulfur cluster biogenesis, and NADPH pool maintenance [43,44,46]. To elucidate mechanisms underlying *pos5Δ’s* ZnS QD HS phenotype, its growth was tested with zinc sulfate (ZnSO_4_), and viability and oxidative stress following exposure to ZnS QDs were assessed via flow cytometry.

#### 3.4.1. Difference Between Exposure to Nano (ZnS QDs) and Ionic (Zn^2+^) Zinc

**Figure 4:**
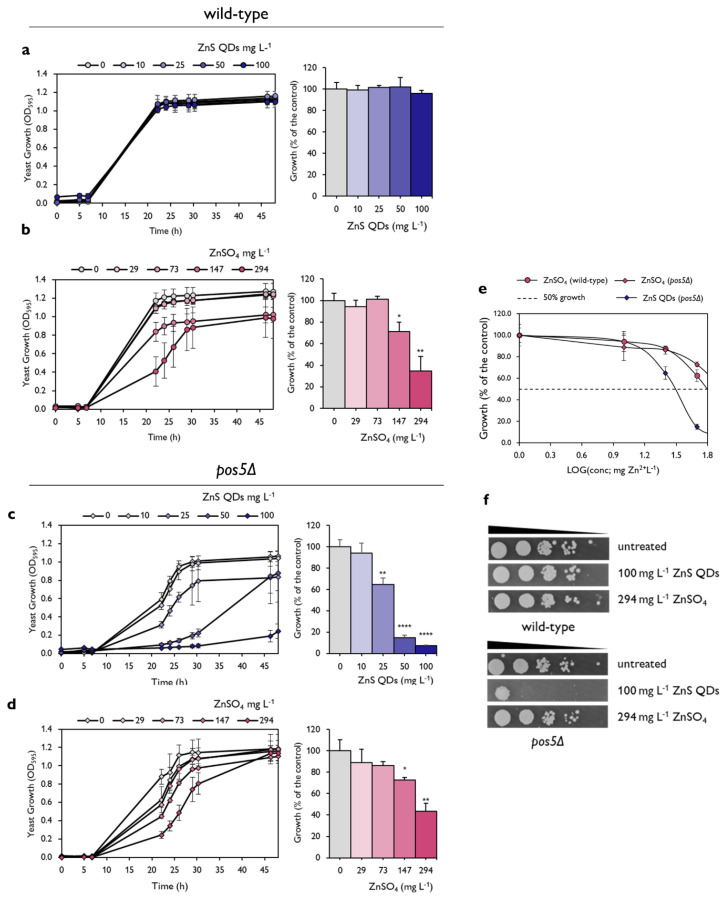
Differential responses of wild-type and *pos5Δ* strains to nano (ZnS QDs) vs. ionic zinc (ZnSO_4_). (**a**–**d**) Cytotoxicity assays in 96-well plates: wild-type (*blue*; **a**,**b**) and *pos5Δ* (*red*; **c**,**d**) exposed to ZnS QDs (10–100 mg L^−1^) or ZnSO_4_ (29–294 mg L^−1^) in YPD. Growth curves (***left***) show optical density over 48 h; histograms (***right***) compare percentage growth at 22 h (wild-type) and 26 h (*pos5Δ*), when untreated cultures exited the exponential phase (Student’s *t*-test; * *p* < 0.05, ** *p* < 0.01, **** *p* < 0.0001; Past 4.12b). (**e**) Dose–response curves for wild-type and *pos5Δ* growth under ZnS QDs or ZnSO_4_. (**f**) Serial dilution spot assays (10^7^–10^3^ cells mL^−1^) of wild-type (***left***) and *pos5Δ* (***right***) on YPD agar ± 100 mg L^−1^ ZnS QDs or 294 mg L^−1^ ZnSO_4_.

To assess the impact of Zn^2+^ eventually released from ZnS QDs, the growth of both the wild-type strain and the *pos5Δ* mutant was evaluated in the presence of zinc sulphate (ZnSO_4_), by serial dilution spot assay in solid medium and by spectrophotometrically monitoring strain growth by increase in optical density in 96-well plates over 48 h (Figure 4). The growth values (in %) during the late exponential phase (untreated control) were compared for all the tested conditions (Figure 4a–d). The tested concentrations of ZnSO_4_ were calculated to match the concentration of Zn present in the core of ZnS QDs. The results obtained on solid medium (Figure 4f) indicated that the release of Zn^2+^ ions from ZnS QDs in the growth medium did not substantially inhibit the growth of both wild-type and *pos5Δ*, but a strong inhibition of *pos5Δ* (and not of the wild-type) was evident in the presence of ZnS QDs. As expected, the growth of the wild-type strain in liquid medium was not affected by the presence of ZnS QDs at the concentrations tested (Figure 4a), while *pos5Δ* mutant exhibited a significant and dose-dependent growth inhibition (Figure 4c), with a 10 h lag at 50 mg L^−1^. Although Zn is an essential micronutrient with crucial roles in various metabolic and physiological processes in *S. cerevisiae* [47], both strains exhibited reduced growth as the concentration of ZnSO_4_ in the growth medium increased to 147 mg L^−1^ (Figure 4b,d); in fact, an excess of Zn in the growth medium increases the formation of reactive oxygen species [48,49]. Dose–response curves (Figure 4e) indicated *pos5Δ*’s 50% growth reduction occurred at lower concentrations of ZnS QDs than of ZnSO_4_, implicating mechanisms beyond Zn^2+^ toxicity. Imperiale et al. (2022) measured the release of Zn and Cd from the relative QDs with the time in suspension and found that only 1.82% and 1.22% of Zn and Cd, respectively, were liberated [50]. For Zn, a small amount would be insufficient to explain *pos5Δ*’s HS phenotype, further supporting ZnS QD-specific effects.

#### 3.4.2. Flow Cytometry Analyses of *pos5Δ* and Wild-Type Exposed to ZnS QDs

A flow cytometry analysis was conducted to determine whether the treatment with ZnS QDs could induce an increase in the formation of reactive oxygen species (ROS) or induce cell death in wild-type and *pos5Δ.* Cells were stained with propidium iodide (PI), which fluoresces upon binding DNA in dead cells, and 2′,7′-dichlorodihydrofluorescein diacetate (DCFH_2_-DA), a non-fluorescent probe that oxidizes to fluorescent DCF in the presence of ROS. After 24 h exposure to 100 mg L^−1^ ZnS QDs, neither strain showed a significant ROS increase (Figure S10, Table S5). While ZnS QDs caused no wild-type cell death, *pos5Δ* showed a 4-fold increase in PI-positive cells. In contrast, prior studies using 100 mg L^−1^ CdS QDs reported ~30% DCF-positive and ~60% PI-positive wild-type cells after 24 h [51]. This highlights ZnS QDs’ lower cytotoxicity compared to CdS QDs in *S. cerevisiae*. Hydrogen peroxide (0.3–3% H_2_O_2_) served as a positive control, inducing ROS-dependent DCF fluorescence in both strains. However, *pos5Δ* exhibited earlier cell death at higher H_2_O_2_ concentrations, consistent with its known oxidative stress sensitivity [43,44]. These results, together with the HS phenotype previously observed in both liquid and solid media, suggest that *pos5Δ* hypersensitivity to ZnS QDs likely stems from combined ROS-independent cytostatic effects or partial oxidative stress exacerbation.

### 3.5. Transcriptional Analyses

Transcriptional changes induced by exposure to ZnS QDs were analyzed by mRNA sequencing of RNA samples extracted from the wild-type strain and from the HS mutant *pos5Δ*. Exposure to ZnS QDs or ZnSO_4_ was utilized to investigate the comparative contribution of the nano and the ionic form of Zn in determining the observed HS phenotype. The analysis was conducted at two time points, 5 and 24 h after treatment, to compare the early and late response. An arbitrary fold-change threshold of 1.5 was used to identify differentially expressed genes (DEGs). The distribution of fold-change values across samples and the number of up- and down-regulated genes, Venn diagrams, PCA, MA, and box plots are reported in Supplementary Materials (Figures S4–S8).

#### 3.5.1. Transcriptional Profile of Wild-Type Exposed to ZnS QDs or ZnSO_4_

**Figure 5:**
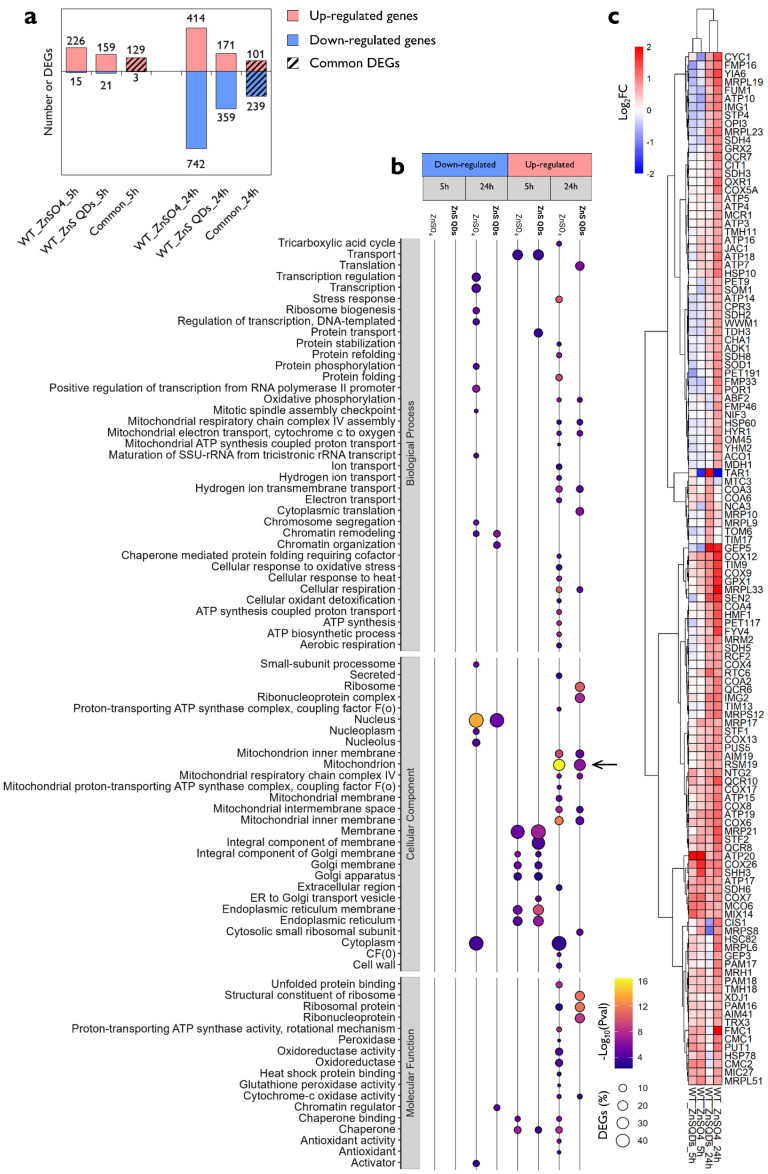
Transcriptional response of wild-type to ZnS QDs and ZnSO_4_. (**a**) Up-regulated and down-regulated genes after 5 h and 24 h treatments. Black bars denote genes regulated in both conditions. (**b**) Enriched annotation terms (Benjamini-adjusted *p* < 0.05) for down-regulated (***left***) and up-regulated (***right***) genes, identified using DAVID and grouped into Biological Process, Cellular Component, and Molecular Function. *p*-value and percentage of associated differentially expressed genes (DEGs) for each annotation term are represented. (**c**) Heatmap of DEGs associated with Cellular Component “Mitochondrion” (*black arrow*) showing differential modulation across treatments. Details on the target genes reported in the heatmap are described in Table S6.

RNA sequencing identified distinct sets of up- and down-regulated genes in wild-type yeast treated with ZnS QDs or ZnSO_4_. Early (5 h) and late (24 h) responses shared 132 (129 up-regulated + 3 down-regulated) and 340 (101 up-regulated + 239 down-regulated) common DEGs, respectively (Figure 5a and Figure S8A). During the early response, both treatments resulted in the up-regulation of genes associated with the endoplasmic reticulum and the Golgi apparatus, and of genes involved in transport processes and chaperone activity. In the late response, a shared up-regulation of genes related to cellular respiration, particularly at the level of the mitochondrial electron transport chain, was observed. At this stage, mitochondria were the most affected cellular compartment. Nevertheless, there were also specific GO terms associated with ZnS QD treatment. In the late response, treatment with ZnS QDs led to a specific up-regulation of translational processes and ribosomal components (Figure 5b). Although several genes encoding mitochondrial-localized proteins (Figure 5c, Table S7) are commonly up-regulated in the late response to nano Zn and ionic Zn, some genes show opposite regulation. Among these, *TAR1*, which encodes a protein involved in regulating respiratory metabolism [52], exhibits QD-specific up-regulation. This suggests that exposure to ZnS QDs, as has previously been observed for CdS QDs [23], may alter the regulation of key mitochondrial functions.

#### 3.5.2. Transcriptional Landscape in *pos5Δ*

Given its role in providing mitochondria with NAPDH reducing power [53], deletion of the *POS5* gene could have pleiotropic effects on gene expression, potentially driving *pos5Δ* to the ZnS QD hypersensitivity phenotype. The *pos5Δ* mutant exhibited an altered transcriptional profile compared to the wild type, with a high number of DEGs both after 5 and 24 h of growth (Figure S11a). Functional annotation (Figure S11b) revealed distinct regulatory patterns: after 5 h of growth, methionine biosynthesis and sulfate assimilation pathways were up-regulated, alongside genes encoding transport proteins and membrane components. Oxidoreductases and chaperones also showed increased expression, a trend persisting into later growth stages, along with a down-regulation of ribosome biogenesis. In a later phase of growth, a down-regulation of protein translation was observed. As expected, there was a significant up-regulation of genes encoding proteins with oxidoreductase activity, many of which are localized in mitochondria, highlighting the importance of the *POS5* gene product in maintaining redox homeostasis in this organelle. Interestingly, copper-binding protein gene expression was elevated even without external stressors, aligning with prior microarray findings of enhanced iron/copper homeostasis gene expression in *pos5Δ* [54]. Although these results are difficult to interpret, the magnitude and diversity of the altered processes underscore a direct and an indirect involvement of the *POS5* gene in various cellular processes.

#### 3.5.3. Transcriptional Profile of *pos5Δ* Exposed to ZnS QDs or ZnSO_4_

**Figure 6:**
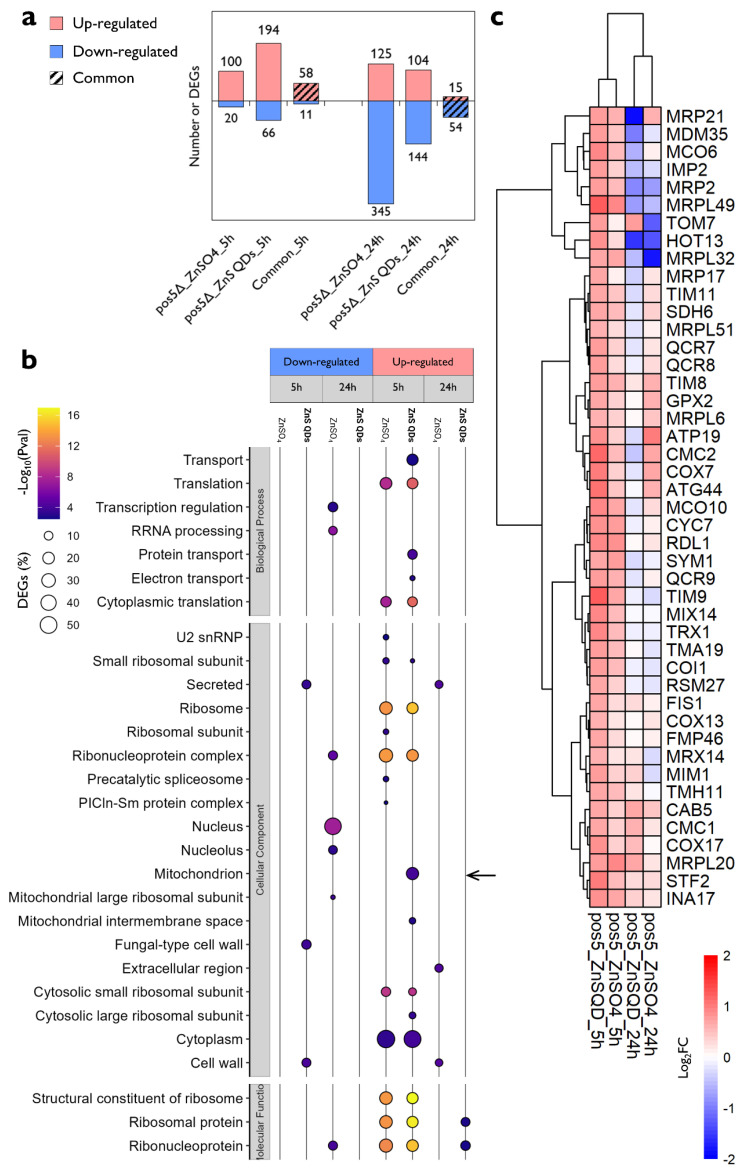
Transcriptional response of *pos5Δ* to ZnS QDs and ZnSO_4_. (**a**) Up-regulated and down-regulated genes after 5 h and 24 h treatments. Black bars denote genes regulated in both conditions. (**b**) Enriched annotation terms (Benjamini-adjusted *p* < 0.05) for down-regulated (***left***) and up-regulated (***right***) genes, identified using DAVID and grouped into Biological Process, Cellular Component, and Molecular Function. *p*-value and percentage of associated differentially expressed genes (DEGs) for each annotation term are represented. (**c**) Heatmap of DEGs associated with Cellular Component “Mitochondrion” (*black arrow*) showing differential modulation across treatments. Details on the target genes reported in the heatmap are described in Table S6.

To elucidate the mechanisms underlying the ZnS QD hypersensitivity phenotype exhibited by *pos5Δ*, RNA-sequencing analysis was performed on RNA samples extracted after 5 and 24 h of exposure to ZnS QDs or ZnSO_4_. Comparative analysis of differentially expressed genes (Figure 6a and Figure S8B) and functional annotation (Figure 6b) revealed shared and unique regulatory patterns. Both treatments induced marked up-regulation of protein synthesis, ribosomes, and ribonucleoprotein complexes. On the other hand, treatment with ZnS QDs resulted in a specific up-regulation of genes involved in protein transport, and of genes encoding proteins with mitochondrial activity and localization. This is an important finding because it shows how the RD (respiratory deficient) condition of *pos5Δ* does not necessarily imply a complete absence of mitochondrial components, but can be possibly related to their noncorrect assembly, function, and organization [18,55]. Regarding the late response, in the case of ZnSO_4_ treatment, there was a down-regulation of transcription and of genes encoding proteins localized in the nucleus. The limited number of terms associated with the treatment with ZnS QDs suggested that effects in *pos5Δ* primarily occurred at an early stage of treatment. Several genes that encode proteins localized in mitochondria are selectively up-regulated as an early response to ZnS QD exposure (Figure 6c, Table S7), including proteins involved in mitochondrial protein transport (*TOM7*, *TIM9*) and in respiratory and oxidative phosphorylation (*CMC2*, *COX7*, *MCO10*), which may lead to an effective mitochondrial adaptation that was totally missing in the *pos5Δ* mutant with the consequent HS phenotype.

#### 3.5.4. Transcriptional Analyses Reveal Differences in Metal Homeostasis Modulation Between Wild-Type and *pos5Δ*

**Figure 7:**
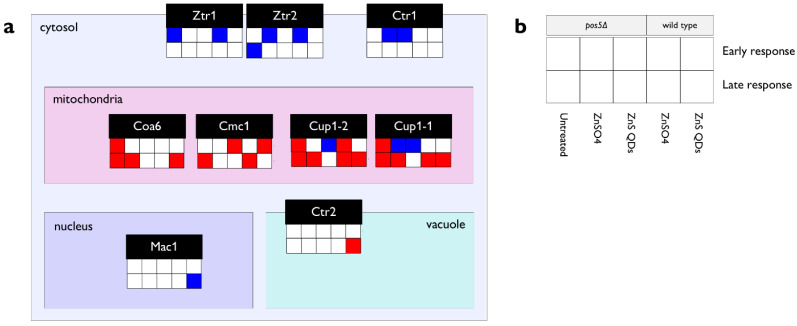
Metal homeostasis gene regulation under ZnS QD or ZnSO_4_ exposure. (**a**) Up-regulated (*red*) and down-regulated (*blue*) genes, categorized by primary cellular localization, exhibit distinct modulation patterns in wild-type and *pos5Δ*. (**b**) Schematic diagram of the displayed samples grouped according to treatment and exposure time. *(Early response*, 5 h treatment; *Late response*, 24 h treatment).

Transcriptional analyses revealed how the modulation of several molecular processes accompanied the exposure to ZnS QDs and ZnSO_4_, differently in the wild-type strain and in the *pos5Δ* mutant. An overview of the modulation patterns of different genes encoding proteins involved in metal homeostasis is reported in Figure 7. Plasma membrane zinc transporters *ZRT1* (high-affinity) and *ZRT2* (low-affinity) [56] were down-regulated in both strains after 5 h ZnSO_4_ exposure, likely due to intracellular ionic zinc accumulation. This response was absent after ZnS QD exposure, supporting their stability and distinct uptake mechanism, potentially via endocytosis as observed for other engineered nanomaterials [57,58] with different physico-chemical properties in terms of stability and intrinsic toxicity, as well as for other types of plastics-based nanomaterials; this might suggest how the internalization process might be related more to a species-specific major way of entry or to a preferential size-dependent mechanism [59]. The absence of endocytosis-related functions in DEGs possibly is justified by the low-level constitutive expression of these functions with their general physiological role [57,58,59]. *pos5Δ* constitutively down-regulated *ZRT1* and *ZRT2* even without ZnSO_4_, reflecting inherent metal homeostasis defects linked to *POS5* deletion [43,54]. Both ZnS QD and ZnSO_4_ treatments altered copper transport genes in wild-type and *pos5Δ*. *CTR1* (regulated by transcription factor Mac1) and *CTR2* (vacuolar copper importer) were modulated, suggesting cross-talk between zinc and copper homeostasis. *COA6*, encoding a mitochondrial intermembrane space protein critical for cytochrome c oxidase assembly [60], was differentially regulated. In wild-type, exposure to ZnS QDs induced a late up-regulation of *COA6,* and the same phenomenon was observed for *pos5Δ* exposed to ZnSO_4_. Interestingly, *pos5Δ* seemed to have constitutively up-regulated *COA6* expression. *CMC1*, encoding a mitochondrial metal chaperone critical for cytochrome c oxidase assembly, was up-regulated early in both strains exposed to ZnS QDs. This suggests ZnS QDs may mimic labile metal pools, triggering compensatory metal-handling responses. Transcriptomic analysis revealed a peculiar modulation pattern of the yeast metallothionein Cup1 across different conditions. While in the wild-type, ZnSO_4_ and ZnS QDs up-regulated metallothionein genes *CUP1-1* and *CUP1-2* after 24 h, untreated *pos5Δ* showed strong up-regulation of *CUP1-1/2*, potentially due to a Cup2 transcription factor binding site in the *POS5* promoter [40]. Notably, treatment with ZnS QDs induced a down-regulation of this metallothionein in *pos5Δ*. In a recent study, Kim and Lindahl (2023) reported that this metallothionein colocalizes in the cytosol and mitochondrial intermembrane space [61]. Although many aspects of its function remain unclear, it was proposed that Cup1 might play a role in limiting the concentration of low-molecular-mass copper within mitochondria [61]. In a recent transcriptomic study conducted by Balfourier et al. (2023), it has been found that, in human cell lines, exposure to Zn-, Cd-, Cu-, or Ag-based NPs, or their corresponding ion forms, induces a strong up-regulation of various metallothioneins, including *MT1X*, *MT2A*, *MT1H*, *MT1E*, and *MT1F* [62]. Defective mitochondrial metal homeostasis in *pos5Δ*, evidenced by disrupted *CUP1* expression, may drive the ZnS QD HS phenotype. This aligns with studies linking metal dysregulation to protein aggregation, endoplasmic reticulum stress, and protein synthesis defects [63,64,65,66]. Globally, this is also evidence with practical implications, as it suggests that ENMs with low intrinsic toxicity (such as ZnS QDs) can become highly toxic in specific genetic backgrounds (e.g., *pos5Δ*) or in the presence of environmental stresses that impair Pos5 activity.

### 3.6. Comparison of ZnS QD and CdS QD Chemogenomic and Transcriptional Evidence

**Figure 8:**
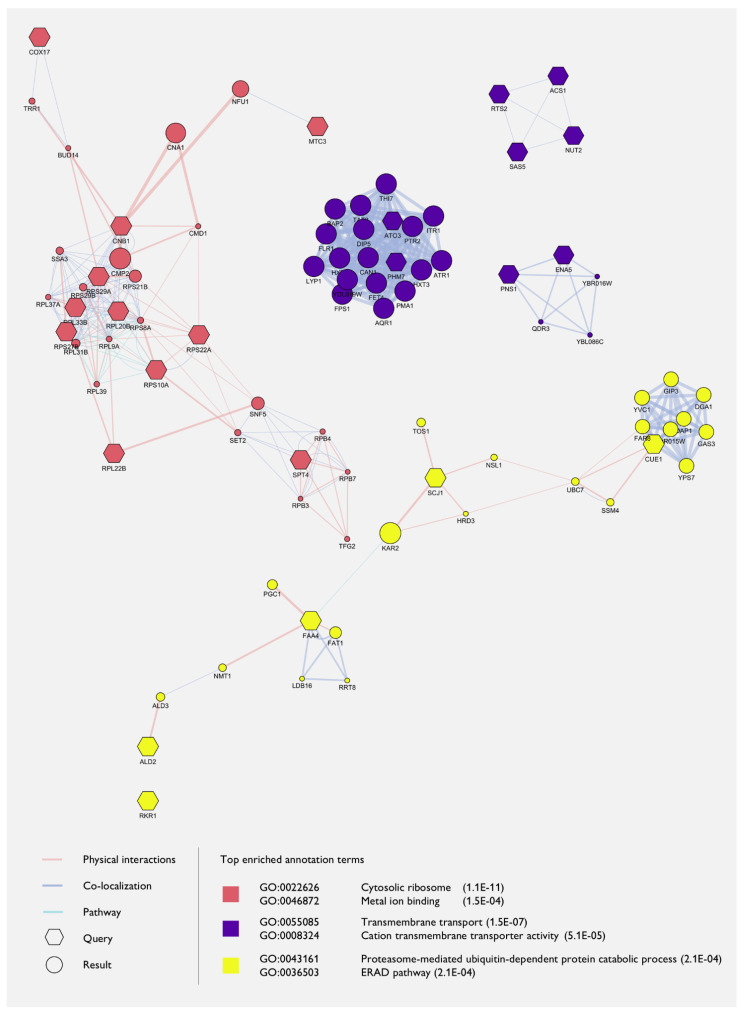
QD-associated response in networks. Network analyses of query genes commonly up-regulated (*red*), down-regulated (*blue*), or harbored by sensitive mutants (*yellow*) identified in both ZnS QD and CdS QD chemogenomic and transcriptomic analyses. The network integrates query genes (*hexagons*) and associated genes (*circles*) with physical interactions, co-localization, pathway associations, and functional annotations (FDR Q-value < 0.05) generated using the GeneMANIA Cytoscape plugin.

Previous studies have applied chemogenomic and transcriptomic approaches to investigate CdS QD toxicity [10,24], and this allows the comparison of genes involved in response to both ZnS QDs and CdS QDs. Globally, 12 commonly up-regulated, 20 down-regulated, and five shared sensitive mutants were identified (Table S8). Network analysis (Figure 8) revealed overlapping targets. Both ZnS and CdS QDs induce the up-regulation of genes involved in protein synthesis and with metal ion binding functions, while a common down-regulation of genes with transmembrane cation transport functions can be observed. Genes deleted in the mutants with double sensitivity are involved in proteasome-mediated ubiquitin-dependent protein catabolism. *scj1Δ*, *rkr1Δ*, and *cue1Δ*, identified in this study among mutants sensitive to ZnS QDs, were previously found to be also sensitive to CdS QDs [10]. *SCJ1* codes for an ER-localized protein that mediates protein folding and maturation in cooperation with Kar2 [67]. *RKR1* and *CUE1* gene products both participate in the ER-associated degradation (ERAD) pathway: Rkr1 is a ubiquitin ligase that targets translationally stalled proteins, while Cue1 mediates misfolded protein ubiquitination [68,69]. In another study relevant to understanding the mechanisms by which the QDs interact with the cell environment, it was shown that QD–protein interaction can promote protein misfolding [26]. In particular, the presence of some HSPs among hard corona proteins can affect cellular proteostasis [26]. Furthermore, several studies have shown that the main effect of exposure of human THP-1 cell lines to CdS QDs is the induction of autophagic pathways [34,70]. Overall, these results suggest that the adverse effects of QDs on metal homeostasis may directly or indirectly interfere with protein stability. More than 60% of the genes identified as primary targets of QD response have homology to human genes (Table S7), highlighting how the study of QD effects even in this simple yeast model can provide scalable information for higher organisms.

## 4. Conclusions

In this study, *Saccharomyces cerevisiae* was used to unravel genetic and molecular mechanisms underlying cellular responses to ZnS QDs, bridging the gap between genotype and phenotype, as a functional biotechnology tool, where the deletion mutant (genotype) become an instrument to highlight the loss of functions (phenotype) during QD exposure. ZnS QDs inhibited wild-type growth only at high concentrations, confirming their lower toxicity compared to CdS QDs. The obtained results evidenced that the mechanism yeast cells display to react in the presence of a low-toxicity nanomaterial (ZnS QDs) is through the activation of a new metal homeostatic regulation and the involvement of metal binding protein, not necessarily specific to Zn. The mitochondrial role in all of these is paramount and confirmed that mitochondria are not solely the “power plant” of the cell but play a fundamental role in modulation of the ENM stress response, at the genetic and epigenetic levels [10,18,23,24].

Screening a yeast knock-out (YKO) collection identified mutants sensitive to ZnS QDs, with mitochondrial functions emerging as a primary target. Further analysis of *sod1Δ*, *glr1Δ,* and the hypersensitive mutant *pos5Δ* revealed mitochondrial redox homeostasis as critical for QD tolerance. Given that humans possess a *POS5* homolog, NADK2, implicated in dienoyl-CoA reductase (DECR) deficiency with hyperlysinemia [71,72], elucidating the role of *POS5* in the response to QDs of biomedical interest is of crucial importance. Transcriptomic profiling of *pos5Δ* showed that ZnS QDs modulate genes involved in intracellular trafficking, electron transport, and metal binding. Notably, the metallothionein genes *CUP1-1* and *CUP1-2* were up-regulated in wild-type cells exposed to ZnS QDs or ZnSO_4_, whereas in *pos5Δ*, where they were constitutively overexpressed, both genes were down-regulated upon treatment. This suggests metallothioneins may mitigate QD toxicity, though their interaction dynamics require further exploration. The constitutive *CUP1* dysregulation in *pos5Δ* highlights the interplay between redox balance and metal homeostasis, suggesting genetic background profoundly influences QD toxicity. The mitochondrial chaperone *COA6* also emerged as a key player, with *coa6Δ* sensitivity and transcriptional induction implicating mitochondrial metal handling in QD response. Comparative analysis revealed shared pathways between ZnS and CdS QDs, particularly in protein synthesis/degradation and metal transport. Future studies should investigate mitochondrial ultrastructural changes and organellar metal distribution post-QD exposure. Additionally, tracking intracellular QD fate in vivo could clarify metallothionein roles.

In general, for different ENMs—and, specifically, for different QD types—toxicity pathways, genetic and epigenetic effects, and cellular response are largely related to the different physico-chemical properties, such as size, surface charge, and compositions, which affect stability, aggregation, and potential interaction within cells (e.g., biofunctionalization, biotransformation) [73,74,75,76]. A comparison can be performed in terms of pathways exploited and cellular components involved, even if effects observed are generally different: effects on amplification/replication of mtDNA, which leads to a copy number increase for ZnS QDs and stoichiometric modifications for CdS QDs [18], and/or to mitochondrial-mediated programmed cell death [34], which were not observed in the case of ZnS QD exposure.

The presence of the cell wall does not limit the direct scalability of nanomaterial internalization mechanisms to human cells, or how *Saccharomyces cerevisiae* reaffirms its versatility as a model organism for the intracellular response targets triggered by QD exposure. This work bridges molecular mechanisms in yeast with broader toxicological principles, providing a framework for assessing nanomaterial safety across biological systems.

## Data Availability

The data presented in this study are available on request from the corresponding author.

## Supplementary Materials

The following supporting information can be downloaded at: https://www.mdpi.com/article/10.3390/nano16120720/s1, Figure S1. Overview of YKO collection screening; Figure S2. TEM image of ZnS QDs; Figure S3. Reads quality assessment; Figure S4. Principal Component Analysis (PCA) of FPKM values across different tested conditions; Figure S5. Box plots with fold-change distribution across different tested conditions; Figure S6. MA plots displaying fold-change values compared with mean expression in wild type strain and pos5Δ treated with ZnS QDs or ZnSO_4_ for 5 or 24 h; Figure S7. Fold-change distribution and number of up- and down-regulated differentially expressed genes in 5 h- and 24 h-treated samples; Figure S8. Venn diagrams with comparison of up-regulated and down-regulated genes across different tested conditions; Figure S9. Vitality of wild-type and selected sensitive mutants exposed to ZnS QDs; Figure S10. Flow cytometry analysis of wild-type and pos5Δ strains following exposure to ZnS QDs; Figure S11. Transcriptional dysregulation in untreated pos5Δ versus wild type; Table S1. Relevant literature for yeast as a model system for quantum dots assessment; Table S2. Physical characterization of the QDs used in the present work; Table S3. List of genes and Gene Ontology classification associated with YKO deletion mutants sensitive to ZnS QDs; Table S4. Yeast GO Slim Mapper annotated terms of genes deleted in ZnS QD-sensitive mutants; Table S5. Percentage value of detected events in each dot plot quadrant in flow cytometry analyses; Table S6. Enriched annotation terms from Functional Annotation Chart; Table S7. List of DEGs associated with Cellular Component “Mitochondrion” modulated across treatments for wild-type and pos5Δ; Table S8. List of genes identified by the comparison of ZnS QDs and CdS QDs chemogenomic and transcriptomic data.
