# Supplementary material for: Phenomic and Transcriptomic Profiling of ZnS QD Response in Saccharomyces cerevisiae: A Quantum Model Organism for a Quantum Dot Study
Source: Nanomaterials (Basel). 2026 Jun 10;16(12):720. doi: 10.3390/nano16120720 (PMC13304748; doi:10.3390/nano16120720)
Supplement: Supplementary file 1 [file nanomaterials-16-00720-s001.zip › nanomaterials-4322372-supplementary.pdf]

Phenomic and Transcriptomic Profiling of ZnS QDs Response in *Saccharomyces cerevisiae*: A Quantum Model Organism for Quantum Dots Study

Sophia Luche [1], Luca Pagano [2], Marta Marmiroli [1] and Nelson Marmiroli [2]

1 Department of Chemistry, Life Sciences and Environmental Sustainability, University of Parma, Parco Area delle Scienze 33/A, 43124 Parma, Italy; sophia.luche@unipr.it (S.L.); marta.marmiroli@unipr.it (M.M.)

2 Consorzio Interuniversitario Nazionale per le Scienze Ambientali (CINSA), University of Parma, Parco Area delle Scienze 11/A, 43124 Parma, Italy; luca.pagano@unipr.it
* Correspondence: nelson.marmiroli@unipr.it

List of Supporting Information

Supplementary Figures

Figure S1. Overview of YKO collection screening.

Figure S2. TEM image of ZnS QDs.

Figure S3. Reads quality assessment.

Figure S4. Principal Component Analysis (PCA) of fragments per kilobase of transcript per million fragments mapped (FPKM) values across different tested conditions**.**

Figure S5. Box plots with fold-change distribution across different tested conditions.

Figure S6. MA plots displaying fold-change values compared with mean expression in wild type strain and *pos5Δ* treated with ZnS QDs or $ZnSO_4$ for 5 or 24 hours

Figure S7. Fold-change distribution and number of up- and down-regulated differentially expressed genes in 5 hour- and 24 hour-treated samples.

Figure S8 Venn diagrams with comparison of up-regulated and down-regulated genes across different tested conditions.

Figure S9. Vitality of wild-type and selected sensitive mutants exposed to ZnS QDs.

Figure S10. Flow cytometry analysis of wild-type and *pos5Δ* strains following exposure to ZnS QDs.

Figure S11. Transcriptional dysregulation in untreated *pos5Δ* versus wild type.

Supplementary Tables

Table S1. Relevant literature for yeast as a model system for quantum dots assessment.

Table S2. Physical characterization of the QDs used in the present work.

Table S3. List of genes and Gene Ontology classification associated with YKO deletion mutants sensitive to ZnS QDs.

Table S4. Yeast GO Slim Mapper annotated terms of genes deleted in ZnS QD-sensitive mutants.

Table S5. Percentage value of detected events in each dot plot quadrant in flow cytometry analyses.

Table S6. Enriched annotation terms from Functional Annotation Chart.

Table S7. List of DEGs associated with Cellular Component “Mitochondrion” modulated across treatments for wild-type and *pos5Δ*.

Table S8. List of genes identified by the comparison of ZnS QDs and CdS QDs chemogenomic and transcriptomic data.

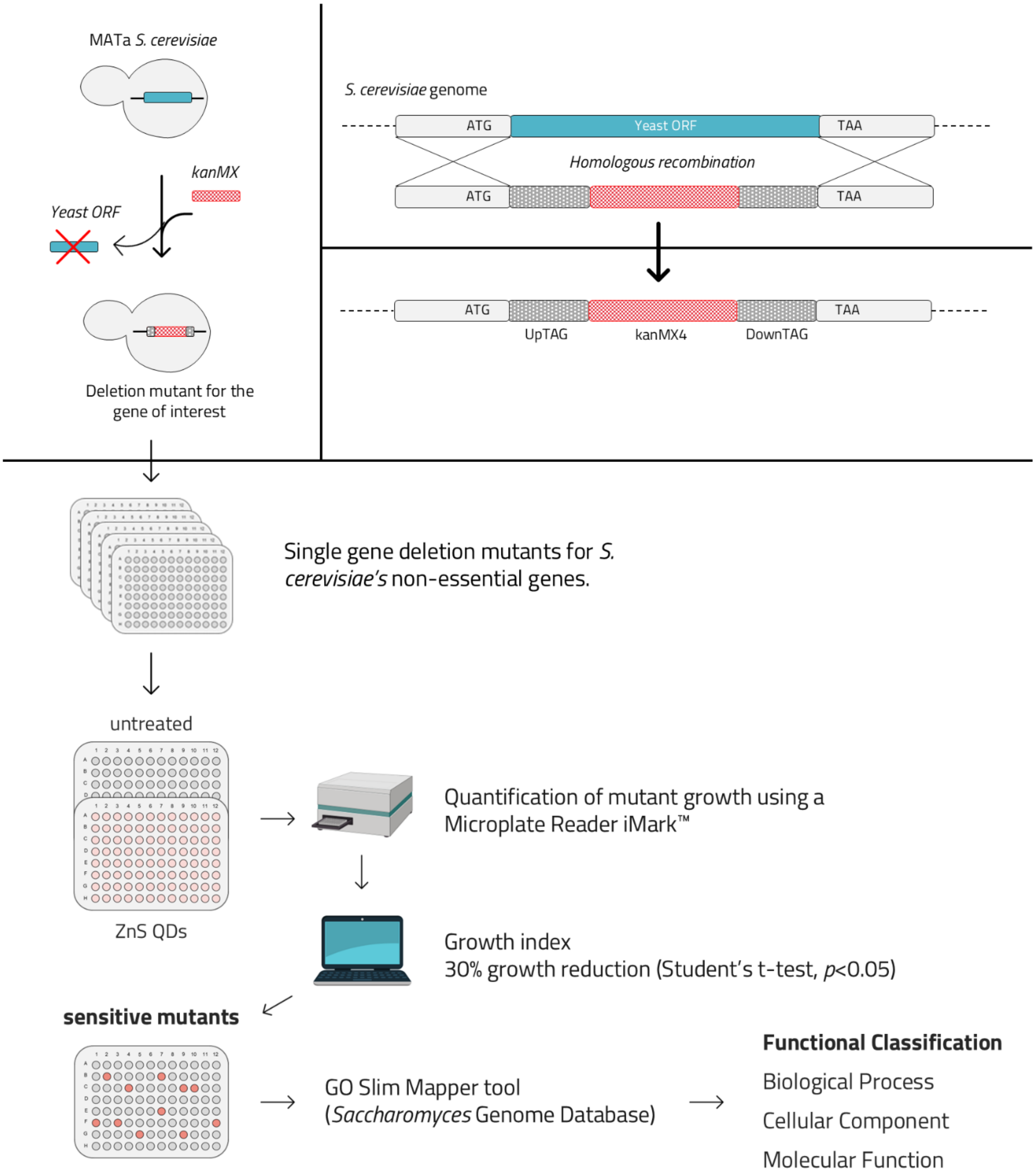

Figure S1. Schematic overview of deletion mutant construction and library screening workflow used to investigate the response of *S. cerevisiae* to ZnS quantum dots.

Table S1. Relevant literature for *Saccharomyces cerevisiae* as a model system for quantum dots and engineered nanomaterials assessment (in citation order).

**Reference**	**num**
Ruotolo R, De Giorgio G, Minato I, Bianchi MG, Bussolati O, Marmiroli N, 2020. Cerium oxide nanoparticles rescue α-synuclein-induced toxicity in a yeast model of parkinson's disease. Nanomaterials 10, 1–18.	7
Käosaar S, Kahru A, Mantecca P, Kasemets K, 2016. Profiling of the toxicity mechanisms of coated and uncoated silver na-noparticles to yeast Saccharomyces cerevisiae BY4741 using a set of its 9 single-gene deletion mutants defective in oxidative stress response, cell wall or membrane integrity and endocyt. Toxicology in Vitro 35, 149–162.	8
Marmiroli M, Pagano L, Pasquali F, Zappettini A, Tosato V, Bruschi C V, Marmiroli N, 2016. A genome-wide nanotoxicology screen of Saccharomyces cerevisiae mutants reveals the basis for cadmium sulphide quantum dot tolerance and sensitivity. Nanotoxicology 10, 84–93.	10
Pasquali F, Agrimonti C, Pagano L, Zappettini A, Villani M, Marmiroli M, White JC, Marmiroli N, 2017. Nucleo-mitochondrial interaction of yeast in response to cadmium sulfide quantum dot exposure. J Hazard Mater 324, 744–752.	23
Pagano L, Caldara M, Villani M, Zappettini A, Marmiroli N, Marmiroli M, 2019. In Vivo-In Vitro Comparative Toxicology of Cadmium Sulphide Quantum Dots in the Model Organism Saccharomyces cerevisiae. Nanomaterials 9.	24
Ruotolo R, Pira G, Villani M, Zappettini A, Marmiroli N, 2018. Ring-shaped corona proteins influence the toxicity of engineered nanoparticles to yeast. Environ Sci Nano 5, 1428–1440.	26
Rossi R, Ruotolo R, De Giorgio G, Marmiroli M, Villani M, Zappettini A, Marmiroli N, 2022. Cadmium Sulfide Quantum Dots Adversely Affect Gametogenesis in Saccharomyces cerevisiae. Nanomaterials 12.	27
Kasemets K, Suppi S, Künnis-Beres K, Kahru A, 2013. Toxicity of CuO nanoparticles to yeast saccharomyces cerevisiae BY4741 wild-type and its nine isogenic single-gene deletion mutants. Chem Res Toxicol 26, 356–367.	28
Smith MR, Boenzli MG, Hindagolla V, Ding J, Miller JM, Hutchison JE, Greenwood JA, Abeliovich H, Bakalinsky AT, 2013. Identification of gold nanoparticle-resistant mutants of Saccharomyces cerevisiae suggests a role for respiratory metabolism in mediating toxicity. Appl Environ Microbiol 79, 728–733.	29
Marmiroli M, Birarda G, Gallo V, Villani M, Zappettini A, Vaccari L, Marmiroli N, Pagano L, 2023. Cadmium Sulfide Quantum Dots, Mitochondrial Function and Environmental Stress: A Mechanistic Reconstruction through In Vivo Cellular Approaches in Saccharomyces cerevisiae. Nanomaterials 13.	35
Gharieb MM, Gadd GM, 2004. Role of glutathione in detoxification of metal(loid)s by Saccharomyces cerevisiae. Biometals 17, 183–188.	41
Wysocki R, Tamás MJ, 2010. How Saccharomyces cerevisiae copes with toxic metals and metalloids. FEMS Microbiol Rev 34, 925–951.	42
Zhang W, Bao S, Fang T, 2016. The neglected nano-specific toxicity of ZnO nanoparticles in the yeast Saccharomyces cerevisiae. Sci Rep 6, 1–11.	48
Gallo V, Srivastava V, Bulone V, Zappettini A, Villani M, Marmiroli N, Marmiroli M, 2020. Proteomic Analysis Identifies Markers of Exposure to Cadmium Sulphide Quantum Dots (CdS QDs). Nanomaterials 10.	51
Ozbek O, O. Ulgen K, Ileri Ercan N, 2021. The Toxicity of Polystyrene-Based Nanoparticles in Saccharomyces cerevisiae Is Associated with Nanoparticle Charge and Uptake Mechanism. Chem Res Toxicol 34, 1055–1068.	58

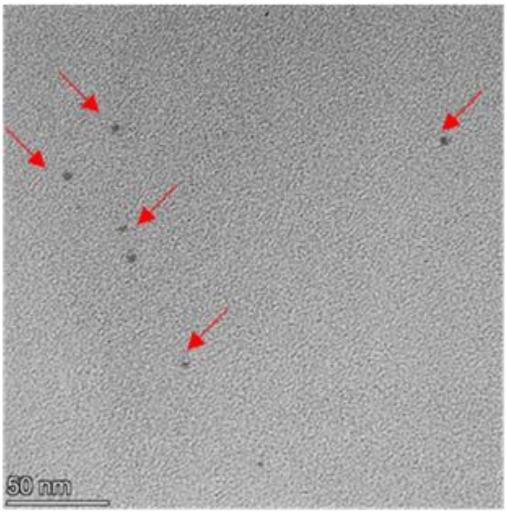

Figure S2. TEM image of ZnS QDs (*red arrows,* scale bar=50 nm).

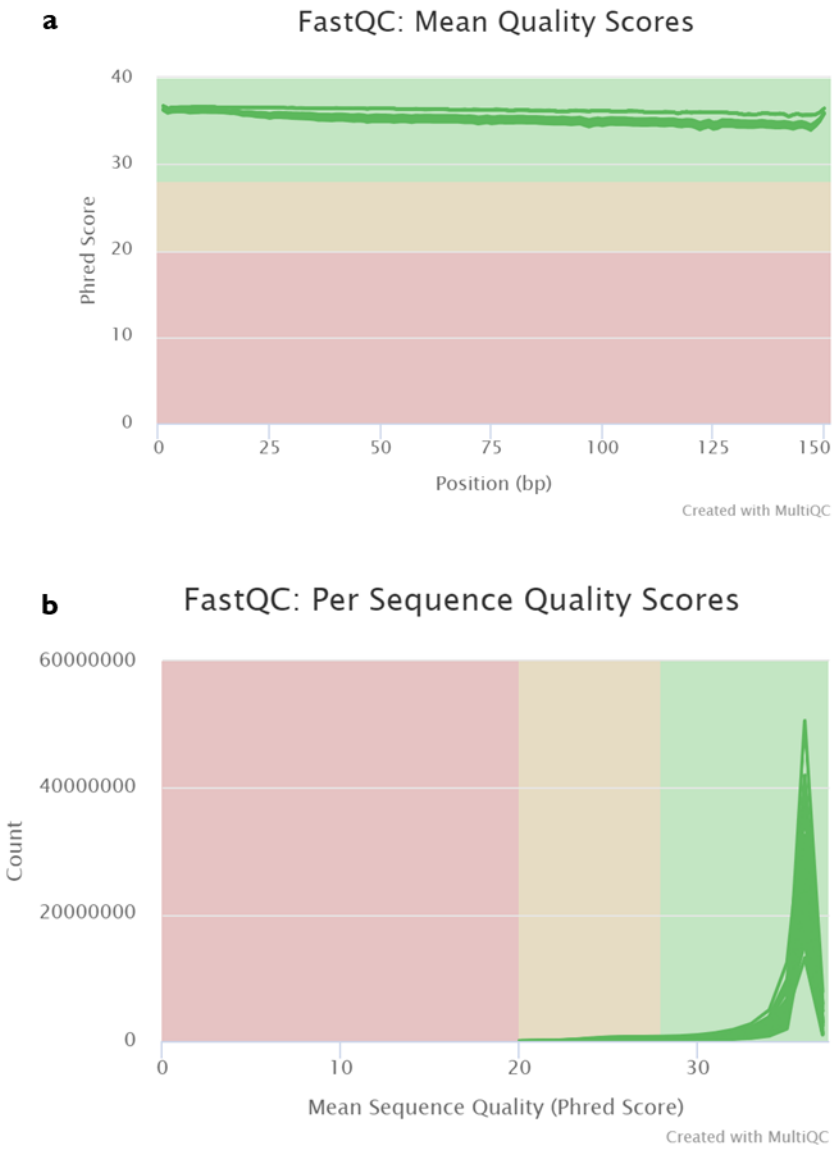

Figure S3. Reads quality assessment. **a**. Mean quality value across each base position in the read. **b**. Number of reads with average quality scores**.**

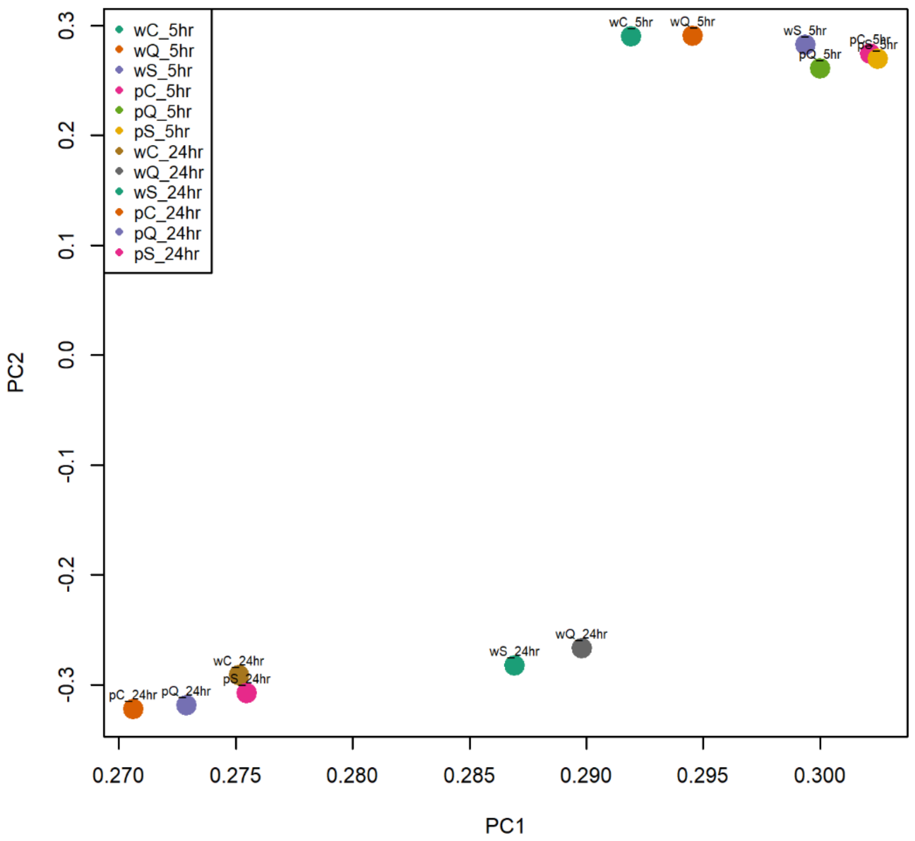

Figure S4. Principal Component Analysis (PCA) of fragments per kilobase of transcript per million fragments mapped (FPKM) values across different tested conditions.

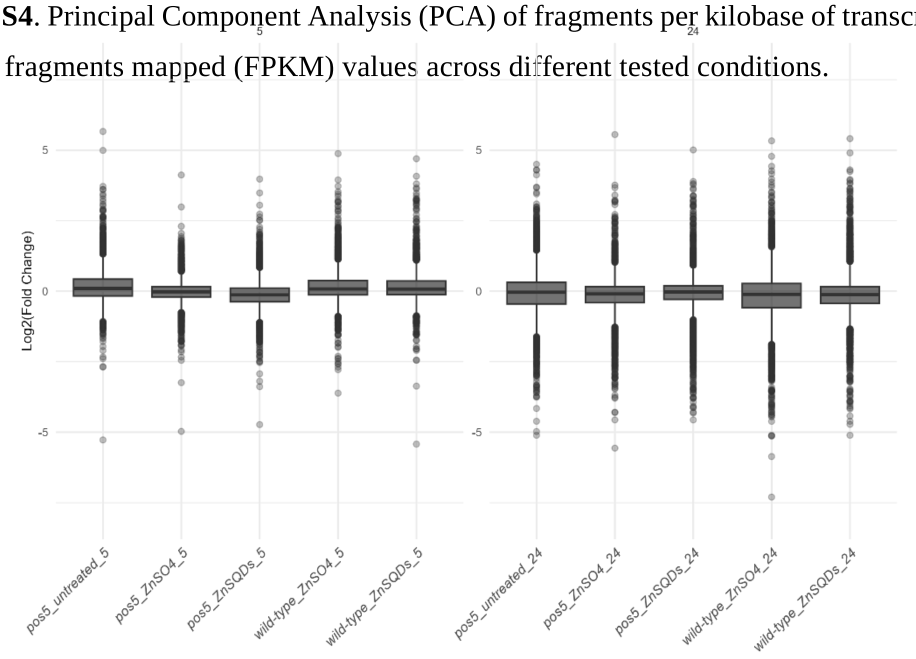

Figure S5. Box plots with fold-change distribution (Log_2 Fold-change) across different tested conditions.

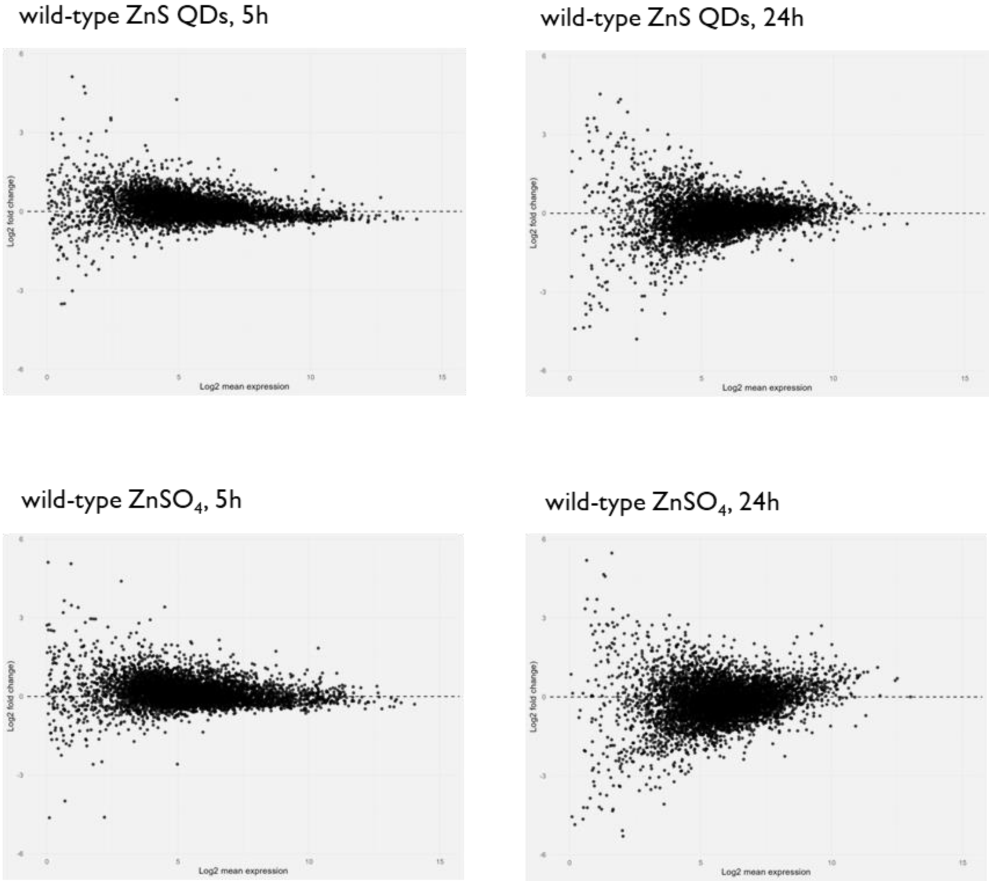

Figure S6. MA plots displaying fold-change values (Log_2 Fold-change) compared with mean expression (Log_2 mean expression) in wild type strain treated with ZnS QDs or $ZnSO_4$ for 5 or 24 hours (normalized on untreated wild type). *(figure continued on next page).*

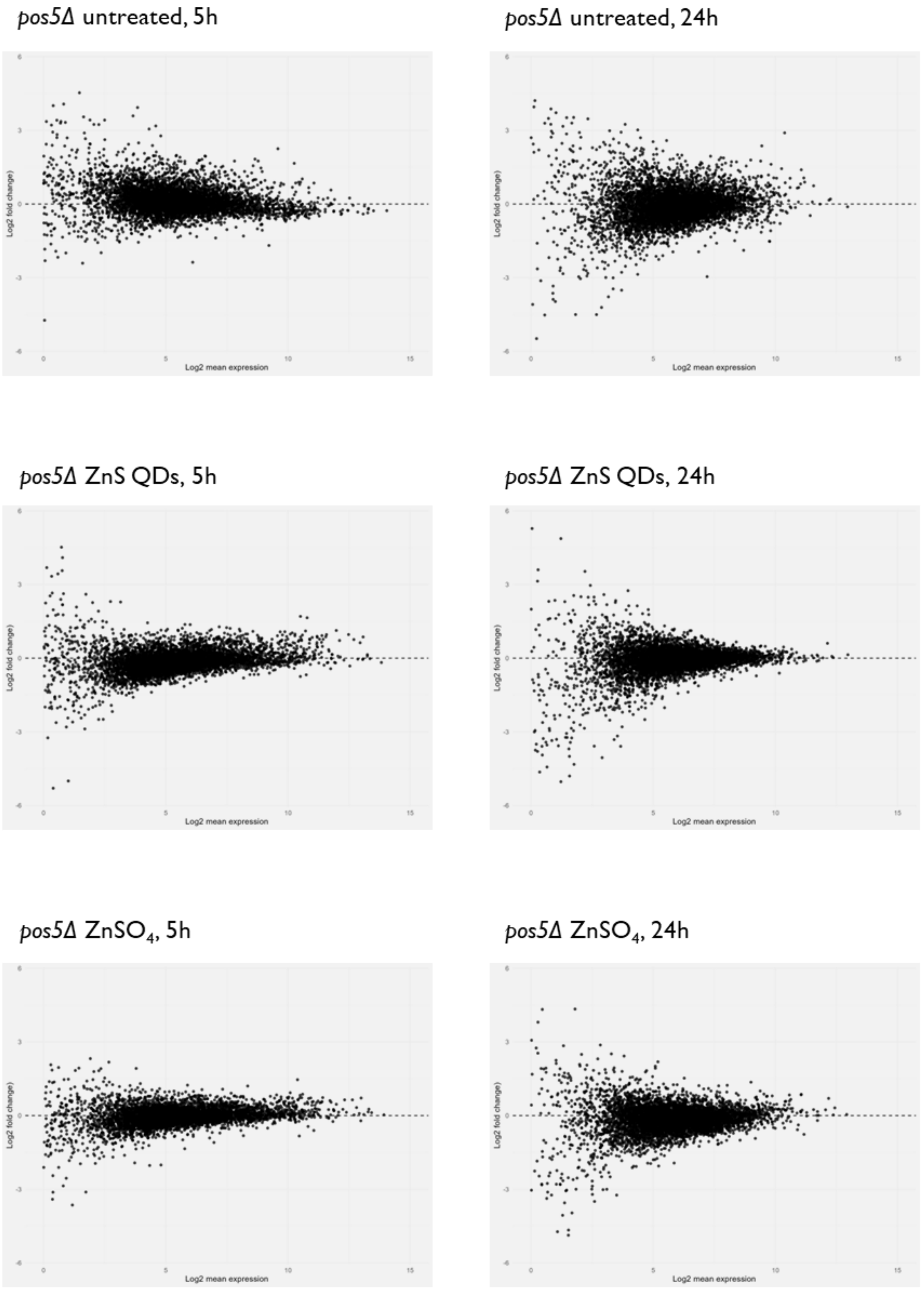

Figure S6. *(continued)*. MA plots of *pos5Δ* transcriptomic response in untreated condition (normalized on untreated wild type) and treated with ZnS QDs or ZnSO$_4$ for 5 or 24 hours (normalized on untreated *pos5Δ*).

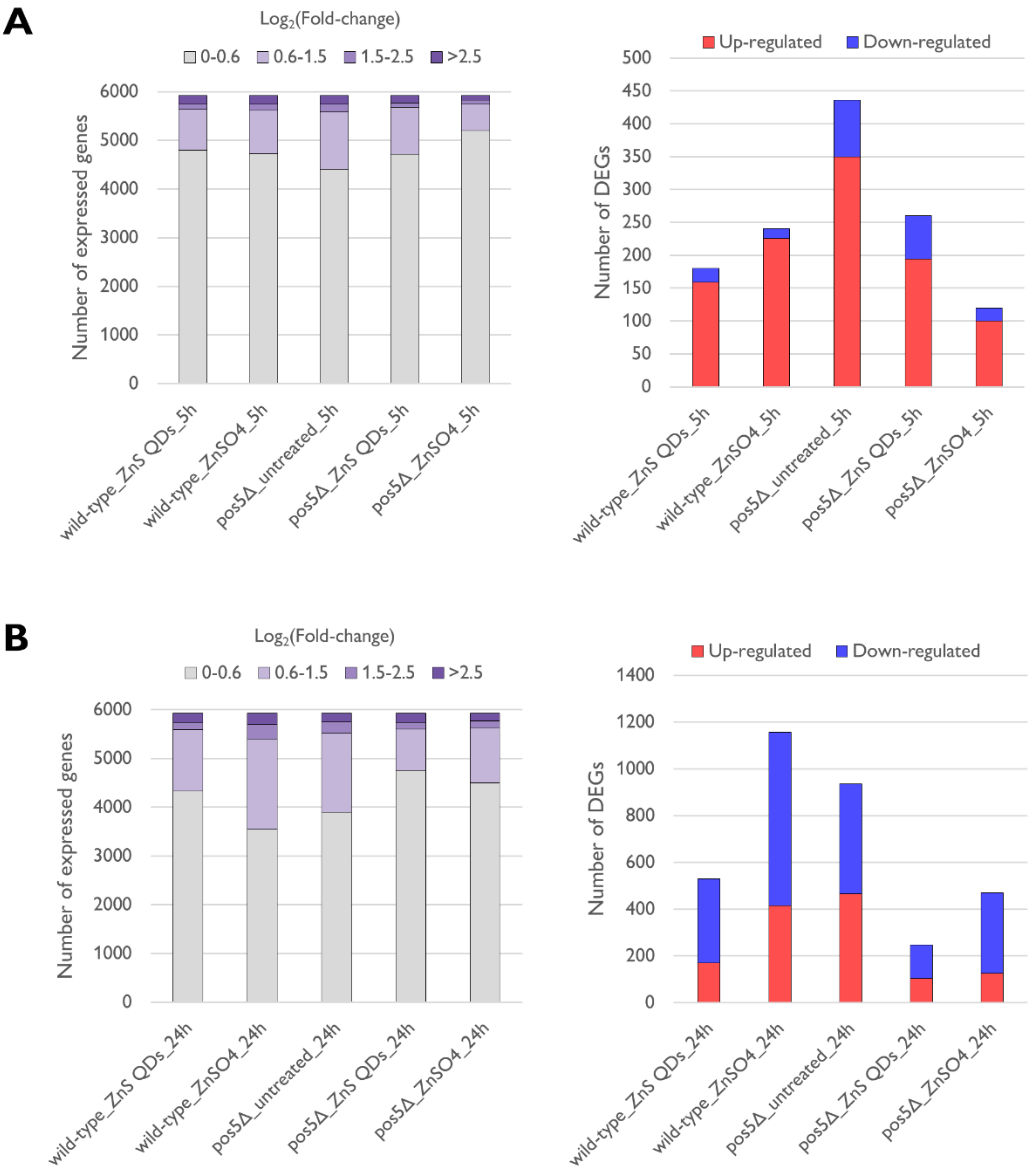

Figure S7. Fold-change distribution (*left*) and number of up- and down-regulated (*right*) differentially expressed genes in 5 hour- (A) and 24 hour- (B) treated samples.

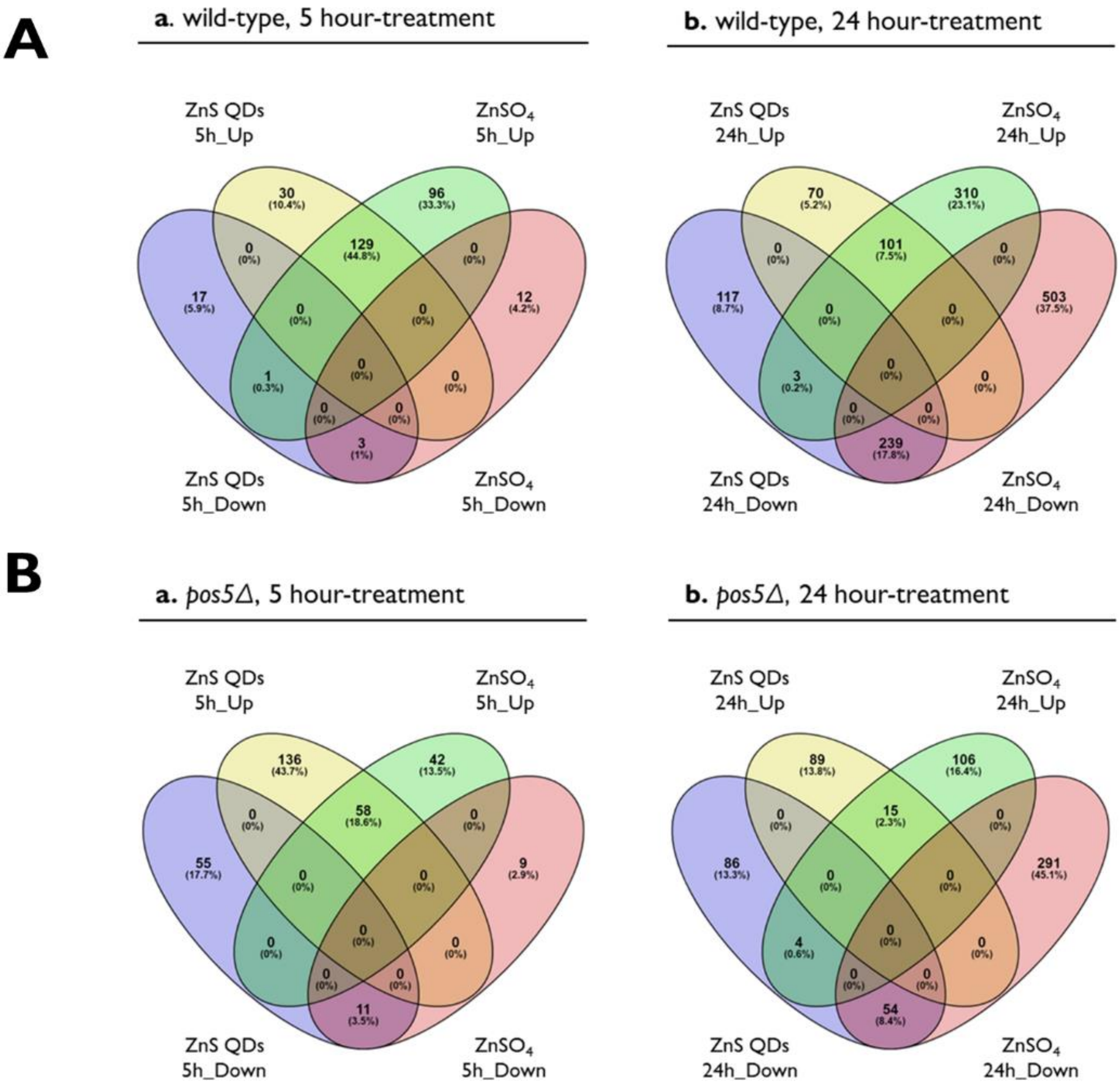

Figure S8. Venn diagrams with a comparison of up-regulated and down-regulated genes identified in wild-type (A) and *pos5Δ* (B) treated with ZnS QDs or $ZnSO_4$ (vs untreated control) after 5 (a) or 24 hours (b). *[Figure continued on next page]*

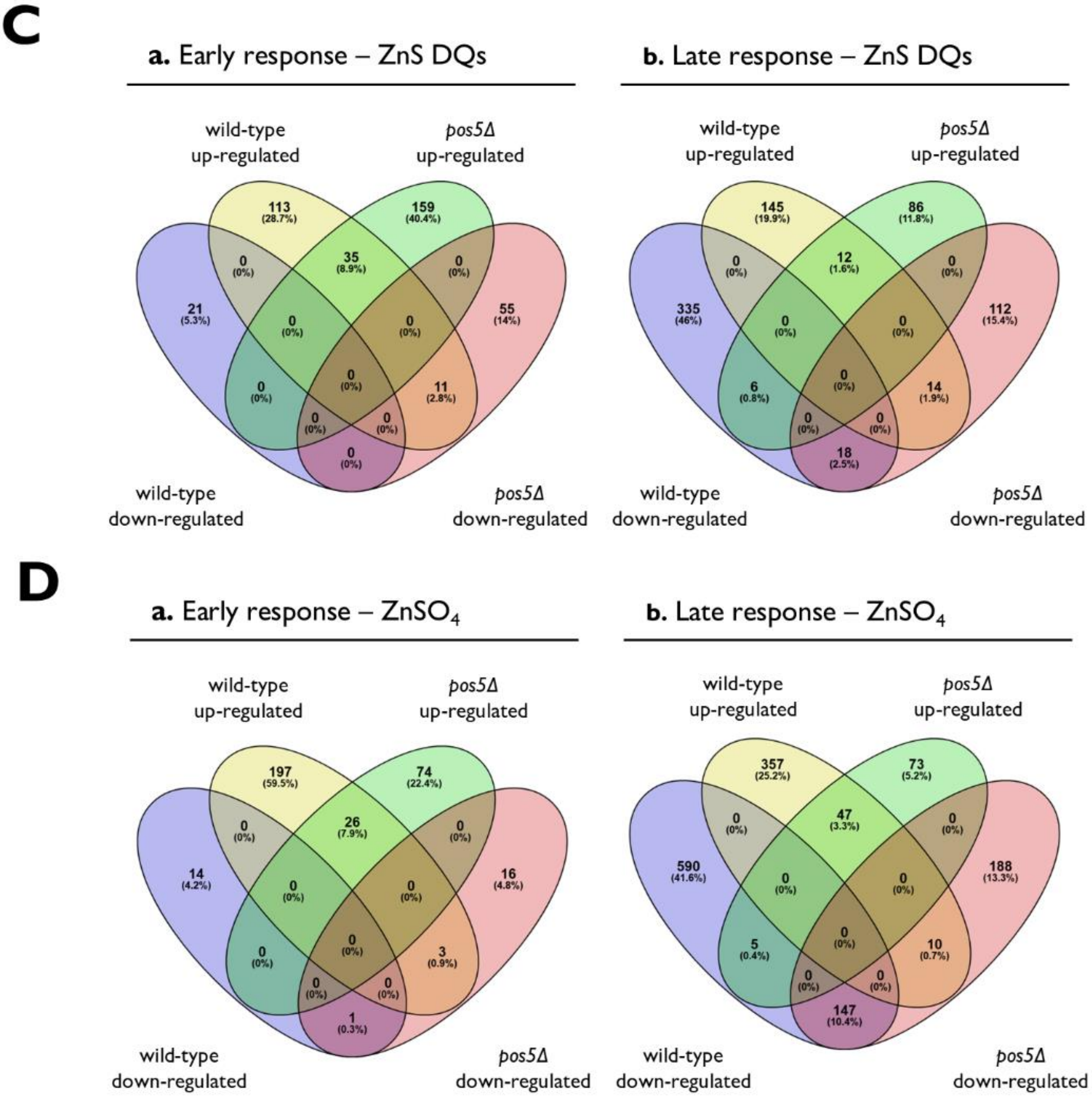

Figure S8. *[continued]* Comparison of genes modulated in wild-type and *pos5Δ* after exposure to ZnS QDs (C) $ZnSO_4$ (D) after 5 (a) or 24 hours (b). Numbers with and without parentheses represent the total number and percentage of genes included in the subset within each subset.

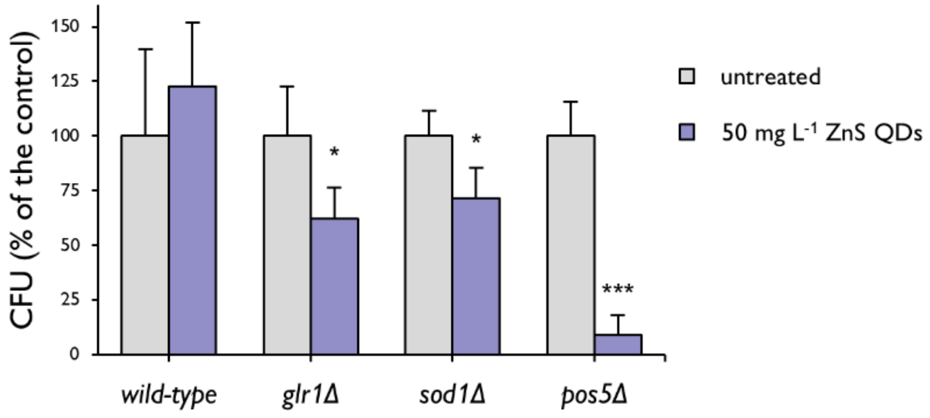

Figure S9. **Vitality of wild-type and selected sensitive mutants exposed to ZnS QDs.** Colony-forming unit (CFU) quantification of yeast after 24-hour exposure to 50 mg L^{-1} ZnS QDs in YPD medium (Student's t-test; *p < 0.05, ***p < 0.001).

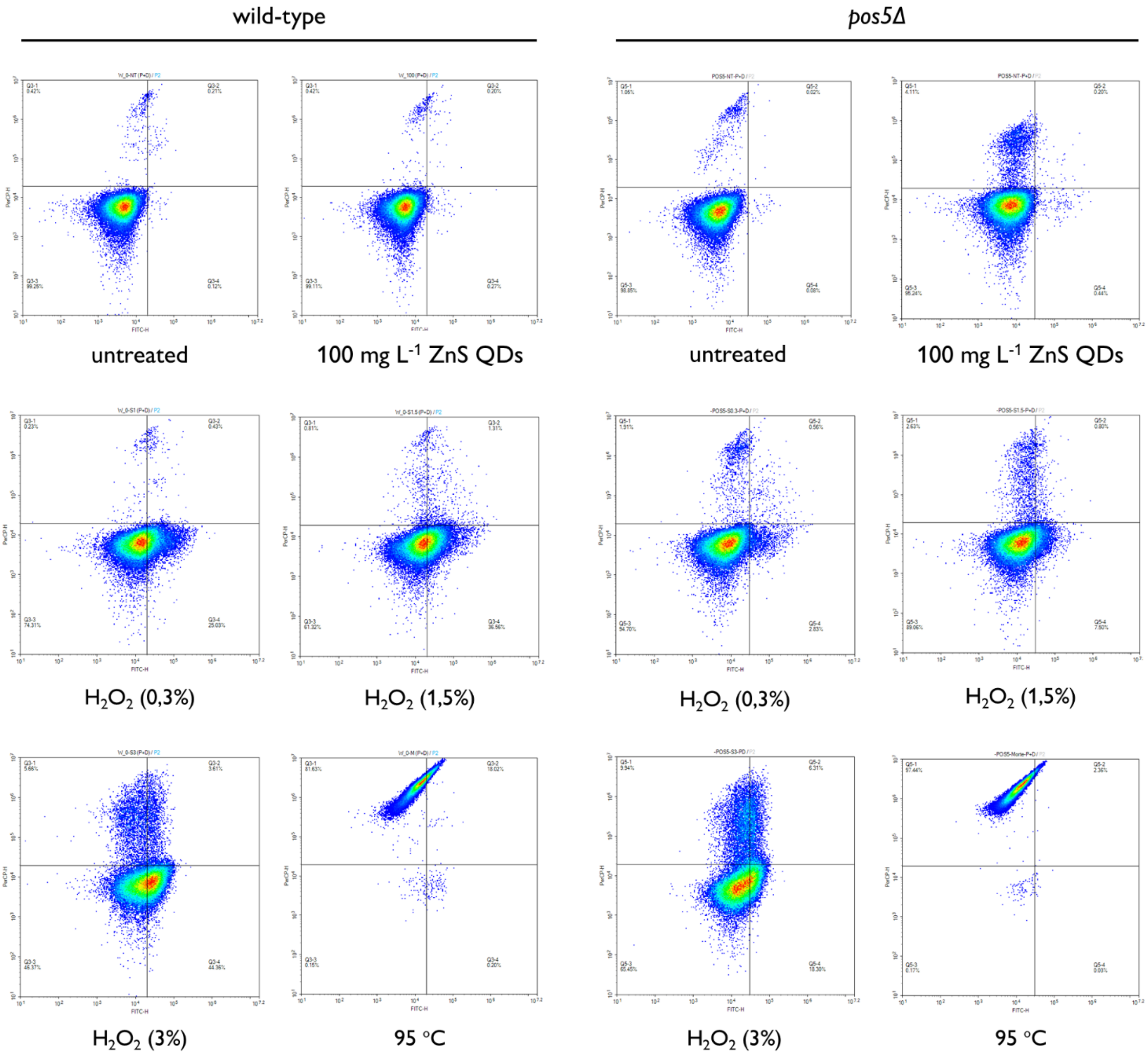

Figure S10. Flow cytometry analysis of wild-type (*left*) and pos5Δ (*right*) strains after 24-hour exposure to 100 mg L^{-1} ZnS QDs in YPD. Cells were stained with $DCFH_2$-DA (FITC channel, ROS detection) and propidium iodide (PI; PerCP channel, dead cells). Positive controls for oxidative stress (0.3 – 3% H_2O_2) and dead cells (95 °C treatment) are reported. (Upper left quadrant (Q1), PI^+/DCF^-, dead cells, no (or low) ROS; upper right quadrant (Q2), PI^+/DCF^+, dead cells, ROS accumulation; lower left quadrant (Q3), PI^-/DCF^-, living cells, no (or low) ROS; lower right quadrant (Q4), PI^-/DCF^+, living cells, ROS accumulation).

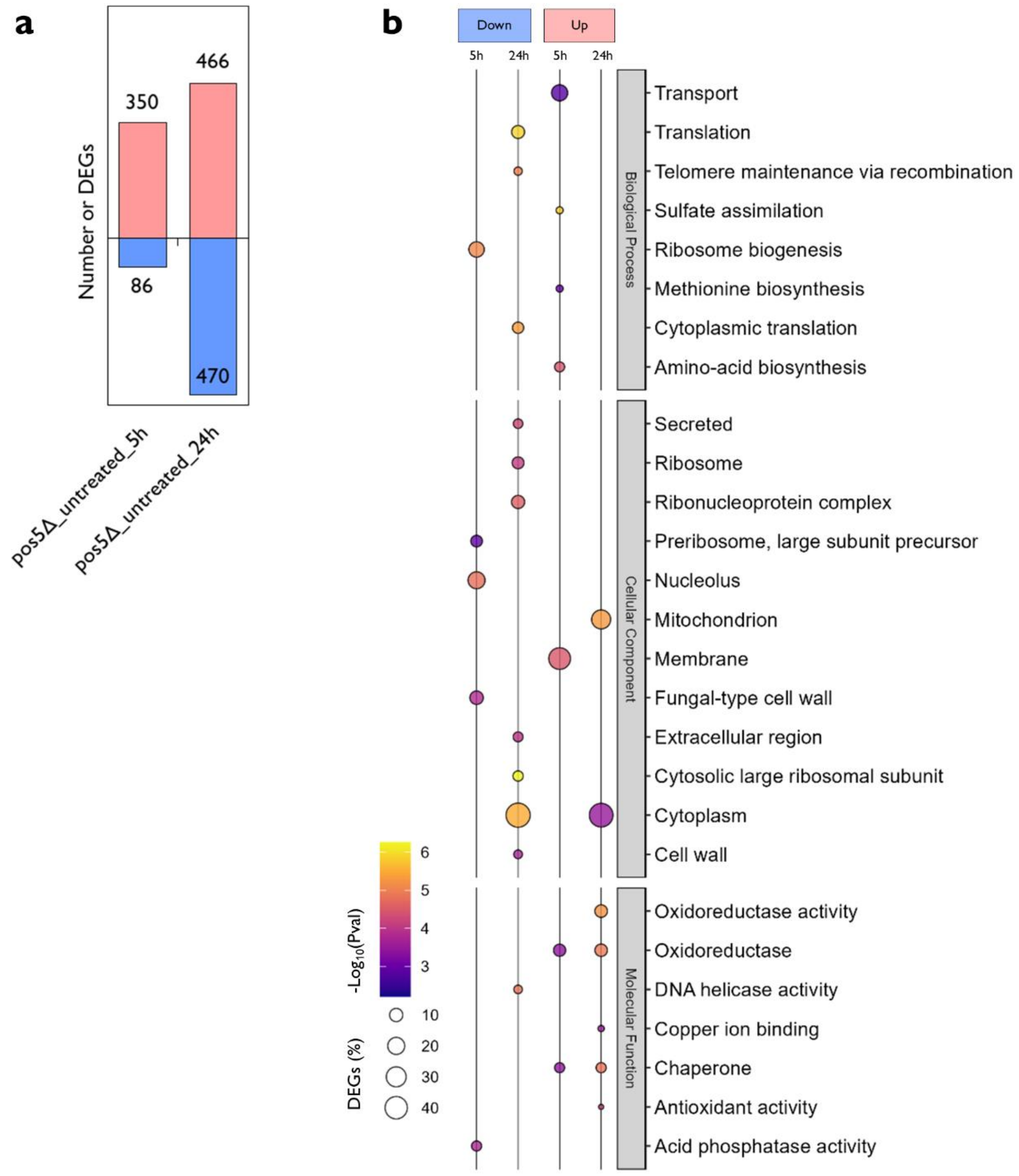

Figure S11. Transcriptional dysregulation in untreated *pos5Δ* versus wild type. (**a**) Number of up-regulated and down-regulated genes in *pos5Δ* after 5 or 24 hours of growth. (**b**) Functional annotation of enriched terms (Benjamini-adjusted $p < 0.05$) for down-regulated (*left*) and up-regulated (*right*) genes at 5 hours and 24 hours. Terms were grouped into three categories (Biological Process, Cellular Component, Molecular Function) using DAVID. P-value and percentage of associated differentially expressed genes (DEGs) for each annotation term are represented.

Table S2. Physical characterization of the QDs used in the present study.

ENMs	Size (nm)	Z-potential (mV)	Hydrodynamic range (nm)
CdS QDs	3-5	-22.0	196.5
ZnS QDs	3-5	+12.0	302.9

Table S3. List of genes and Gene Ontology classification associated with YKO deletion mutants sensitive to ZnS QDs (YeastMine, *Saccharomyces* Genome Database).

Systematic Name	Gene Standard Name	Gene Description	GO category	Ontology Term
YMR170C	*ALD2*	Cytoplasmic aldehyde dehydrogenase; involved in ethanol oxidation and beta-alanine biosynthesis; uses NAD+ as the preferred coenzyme; expression is stress induced and glucose repressed; very similar to Ald3p	*biological_process*	polyamine catabolic process<br>nitrogen compound metabolic process<br>pantothenate biosynthetic process<br>beta-alanine biosynthetic process
			cellular_component	cytoplasm
			molecular_function	aldehyde dehydrogenase (NAD+) activity<br>oxidoreductase activity<br>oxidoreductase activity, acting on the aldehyde or oxo group of donors, NAD or NADP as acceptor<br>glyceraldehyde-3-phosphate dehydrogenase (NAD+) (non-phosphorylating) activity
YNL315C	*ATP11*	Molecular chaperone; required for the assembly of alpha and beta subunits into the F1 sector of mitochondrial F1F0 ATP synthase; N-terminally propionylated in vivo	*biological_process*	mitochondrion organization<br>mitochondrial proton-transporting ATP synthase complex assembly<br>protein-containing complex assembly
			cellular_component	mitochondrion
			molecular_function	unfolded protein binding
YMR244C-A	*COA6*	Protein involved in cytochrome c oxidase (Complex IV) assembly; involved in delivery of copper to Complex IV; also required for efficient formation of respiratory supercomplexes comprised of Complexes III and IV; localizes to the mitochondrial intermembrane space; ortholog implicated in cardiac defects in zebrafish and human; transcription is induced in response to the DNA-damaging agent MMS; protein abundance	*biological_process*	intracellular copper ion homeostasis<br>mitochondrial cytochrome c oxidase assembly
			cellular_component	nucleus<br>cytoplasm<br>mitochondrion<br>mitochondrial intermembrane space<br>respiratory chain complex IV
			disease_ontology	cardiomyopathy<br>COX deficiency, infantile mitochondrial myopathy<br>cytochrome-c oxidase deficiency disease
			molecular_function	copper ion binding

		increases in response to DNA replication stress		
YMR264W	*CUE1*	Ubiquitin-binding protein; ER membrane protein that recruits and integrates the ubiquitin-conjugating enzyme Ubc7p into ER membrane-bound ubiquitin ligase complexes that function in the ER-associated degradation (ERAD) pathway for misfolded proteins; contains a CUE domain that binds ubiquitin to facilitate intramolecular monoubiquitination and to promote diubiquitin elongation, facilitating polyubiquitin chain formation	*biological_process*	ERAD pathway<br>establishment of protein localization to endoplasmic reticulum membrane
			cellular_component	Doa10p ubiquitin ligase complex<br>Hrd1p ubiquitin ligase ERAD-L complex<br>mitochondrion<br>endoplasmic reticulum<br>endoplasmic reticulum membrane<br>membrane
			molecular_function	ubiquitin binding<br>ubiquitin-protein transferase activator activity
YMR160W	*CVM1*	Protein involved in sphingolipid metabolism; localizes to vacuolar contact sites with mitochondria, ER and peroxisomes; mutant has enhanced sensitivity to overexpression of mutant huntingtin; relative distribution within the vacuolar membrane changes upon DNA replication stress	*biological_process*	sphingolipid metabolic process
			cellular_component	fungal-type vacuole membrane<br>peroxisomal membrane<br>endoplasmic reticulum-vacuole membrane contact site<br>vacuole-mitochondrion membrane contact site
			molecular_function	molecular_function
YMR173W	*DDR48*	DNA damage-responsive protein; expression is increased in response to heat-shock stress or treatments that produce DNA lesions; contains multiple repeats of the amino acid sequence NNNDSYGS; protein	*biological_process*	DNA repair
			cellular_component	cytoplasm<br>cytosol
			molecular_function	GTPase activity<br>ATP hydrolysis activity

		abundance increases in response to DNA replication stress		
YMR162C	*DNF3*	Trans-Golgi network aminophospholipid translocase (flippase); type 4 P-type ATPase; involved in phospholipid translocation, contributing to the maintenance of membrane lipid asymmetry in post-Golgi secretory vesicles; role in protein trafficking between the Golgi and endosomal system; localizes to the trans-Golgi network; localizes to the shmoo tip where it has a redundant role in the cellular response to mating pheromone	*biological_process*	lipid transport<br>post-Golgi vesicle-mediated transport<br>pseudohyphal growth<br>phospholipid transport<br>endocytic recycling<br>phospholipid translocation<br>nitrogen compound transport<br>aminophospholipid translocation
			cellular_component	endosome<br>endoplasmic reticulum<br>Golgi apparatus<br>trans-Golgi network<br>plasma membrane<br>endosome membrane<br>membrane<br>trans-Golgi network transport vesicle<br>mating projection tip membrane
			molecular_function	nucleotide binding<br>magnesium ion binding<br>ATP binding<br>ATP hydrolysis activity<br>metal ion binding<br>phosphatidylcholine floppase activity<br>phosphatidylethanolamine flippase activity<br>ATPase-coupled intramembrane lipid transporter activity<br>glycerophospholipid flippase activity<br>phosphatidylcholine flippase activity<br>phosphatidylserine flippase activity
YMR246W	*FAA4*	Long chain fatty acyl-CoA synthetase; activates fatty acids with a preference for C12:0-C16:0 chain lengths; role in the competitive import of long-chain fatty acids and sphingoid long-chain bases; role in stationary phase survival; localizes to lipid particles and the plasma membrane; role	*biological_process*	long-chain fatty acid metabolic process<br>lipid metabolic process<br>fatty acid metabolic process<br>long-chain fatty-acyl-CoA metabolic process<br>long-chain fatty acid import into cell<br>sphingoid long-chain base transport
			cellular_component	cytoplasm<br>endoplasmic reticulum<br>lipid droplet<br>plasma membrane<br>cytoplasmic stress granule

		in sphingolipid-to-glycerolipid metabolism; forms cytoplasmic foci upon replication stress; faa1 faa4 double null complemented by any of human ACSBG1, ACSL1, 3, 4, 5, 6, SLC27A2, or 4	*disease_ontology*	Sjogren-Larsson syndrome
			molecular_function	nucleotide binding<br>long-chain fatty acid-CoA ligase activity<br>ATP binding<br>ligase activity
YMR232W	*FUS2*	Cell fusion regulator; cytoplasmic protein localized to shmoo tip; required for alignment of parental nuclei before nuclear fusion during mating; contains a Dbl-homology domain; binds specifically with activated Cdc42p	*biological_process*	karyogamy involved in conjugation with cellular fusion<br>conjugation with cellular fusion<br>cytogamy<br>regulation of actomyosin contractile ring contraction<br>regulation of division septum assembly
			cellular_component	nucleus<br>cytoplasm<br>mitochondrion<br>mating projection tip<br>cell tip
			molecular_function	molecular_function<br>guanyl-nucleotide exchange factor activity
YMR307W	*GAS1*	Beta-1,3-glucanosyltransferase required for cell wall assembly; also has role in transcriptional silencing; localizes to cell surface via glycosylphosphatidylinositol (GPI) anchor, also found at nuclear periphery; genetic interactions with histone H3 lysine acetyltransferases GCN5 and SAS3 indicate functions for Gas1p in DNA damage response and cell cycle regulation; protein demonstrates amyloid properties in vivo	*biological_process*	filamentous growth<br>fungal-type cell wall organization<br>heterochromatin formation<br>cell wall organization<br>cellular component organization or biogenesis<br>fungal-type cell wall organization or biogenesis<br>fungal-type cell wall (1->3)-beta-D-glucan biosynthetic process<br>regulation of response to endoplasmic reticulum stress
			cellular_component	primary cell septum<br>extracellular region<br>cellular bud scar<br>mitochondrion<br>plasma membrane<br>fungal-type cell wall<br>membrane<br>COPII-coated ER to Golgi transport vesicle<br>nuclear periphery<br>membrane raft<br>side of membrane
			molecular_function	transferase activity<br>glucanosyltransferase activity

				1,3-beta-glucanosyltransferase activity
YMR255W	*GFD1*	Coiled-coiled protein of unknown function; identified as a high-copy suppressor of a dbp5 mutation; protein abundance increases in response to DNA replication stress	*biological_process*	mRNA export from nucleus<br>protein transport<br>mRNA transport
			cellular_component	nucleus<br>nuclear pore<br>cytoplasm<br>membrane<br>nuclear membrane
			molecular_function	protein-containing complex binding
YPL091W	*GLR1*	Cytosolic and mitochondrial glutathione oxidoreductase; converts oxidized glutathione to reduced glutathione; cytosolic Glr1p is the main determinant of the glutathione redox state of the mitochondrial intermembrane space; mitochondrial Glr1p has a role in resistance to hyperoxia; also detected in peroxisomes; protein abundance increases in response to DNA replication stress	*biological_process*	glutathione metabolic process<br>protein glutathionylation<br>cellular response to oxidative stress<br>cell redox homeostasis<br>cellular oxidant detoxification
			cellular_component	nucleus<br>cytoplasm<br>mitochondrion<br>peroxisome<br>cytosol
			disease_ontology	limb-girdle muscular dystrophy
			molecular_function	glutathione-disulfide reductase (NADP) activity<br>oxidoreductase activity<br>oxidoreductase activity, acting on a sulfur group of donors, NAD(P) as acceptor<br>flavin adenine dinucleotide binding<br>NADP binding
YJL142C	*IRC9*	Putative protein of unknown function; partially overlaps verified gene YAK1/YJL141C but does not share all phenotypes; null mutant displays increased levels of spontaneous Rad52p foci, increased sporulation efficiency, and small defect in vacuolar fragmentation	*biological_process*	mitotic recombination<br>replication-born double-strand break repair via sister chromatid exchange
			cellular_component	cellular_component<br>membrane
			molecular_function	molecular_function
YOL103W	*ITR2*	Myo-inositol transporter; member of the sugar transporter superfamily;	*biological_process*	polyol transmembrane transport<br>myo-inositol transport<br>transmembrane transport

		expressed constitutively; ITR2 has a paralog, ITR1, that arose from the whole genome duplication		myo-inositol import across plasma membrane
			cellular_component	fungal-type vacuole<br>plasma membrane<br>membrane<br>cell periphery
			molecular_function	myo-inositol transmembrane transporter activity<br>myo-inositol:proton symporter activity<br>transmembrane transporter activity
YDR033W	*MRH1*	Protein that localizes primarily to the plasma membrane; also found at the nuclear envelope; long-lived protein that is asymmetrically retained in the plasma membrane of mother cells; the authentic, non-tagged protein is detected in mitochondria in a phosphorylated state; null mutation confers sensitivity to acetic acid	*biological_process*	biological_process
			cellular_component	mitochondrion<br>endoplasmic reticulum<br>plasma membrane<br>cellular bud<br>membrane
			molecular_function	molecular_function
YNL122C	*MRP35*	Mitochondrial ribosomal protein of the large subunit; homologous to bacterial L35 and human MRPL35 ribosomal proteins	*biological_process*	translation<br>mitochondrial translation
			cellular_component	mitochondrion<br>mitochondrial large ribosomal subunit<br>ribosome<br>large ribosomal subunit<br>ribonucleoprotein complex
			molecular_function	structural constituent of ribosome
YMR164C	*MSS11*	Transcription factor; involved in regulation of invasive growth and starch degradation; controls the activation of FLO11 and STA2 in response to nutritional signals; forms a heterodimer with Flo8p that interacts with the Swi/Snf complex during transcriptional activation of FLO1, FLO11, and STA1	*biological_process*	regulation of cellular biosynthetic process<br>positive regulation of transcription by RNA polymerase II<br>regulation of primary metabolic process
			cellular_component	nucleus<br>cytoplasm
			molecular_function	molecular_function

YMR285C	*NGL2*	Protein involved in 5.8S rRNA processing; Ccr4p-like RNase required for correct 3'-end formation of 5.8S rRNA at site E; similar to Ngl1p; NGL2 has a paralog, NGL3, that arose from the whole genome duplication	*biological_process*<br>*cellular_component*<br><br>*molecular_function*	rRNA processing<br>nucleus<br>cytoplasm<br>RNA binding<br>catalytic activity<br>nuclease activity<br>RNA endonuclease activity<br>exonuclease activity<br>poly(A)-specific ribonuclease activity<br>hydrolase activity<br>hydrolase activity, acting on ester bonds
YPL188W	*POS5*	Mitochondrial NADH kinase; phosphorylates NADH; also phosphorylates NAD(+) with lower specificity; required for the response to oxidative stress	*biological_process*<br><br><br><br><br>*cellular_component*<br><br>*molecular_function*	NADP biosynthetic process<br>iron-sulfur cluster assembly<br>phosphorylation<br>NAD metabolic process<br>cellular response to oxidative stress<br>mitochondrion<br>mitochondrial matrix<br>nucleotide binding<br>NAD+ kinase activity<br>ATP binding<br>kinase activity<br>transferase activity<br>phosphotransferase activity, alcohol group as acceptor<br>NADH kinase activity
YPL148C	*PPT2*	Phosphopantetheine:protein transferase (PPTase); activates mitochondrial acyl carrier protein (Acp1p) by phosphopantetheinylation	*biological_process*<br><br><br><br>*cellular_component*<br>*molecular_function*	lipid metabolic process<br>fatty acid metabolic process<br>fatty acid biosynthetic process<br>holo-[acyl-carrier-protein] biosynthetic process<br>mitochondrion<br>magnesium ion binding<br>holo-[acyl-carrier-protein] synthase activity<br>transferase activity
YPR115W	*RGC1*	Putative regulator of the Fps1p glycerol channel; multiply phosphorylated by Hog1p under osmotic stress; contains a pleckstrin homology domain; forms homodimers and heterodimerizes with	*biological_process*<br><br>*cellular_component*<br>*molecular_function*	cell cycle<br>positive regulation of glycerol transport<br>cytoplasm<br>channel regulator activity

		paralog Ask10p; green fluorescent protein (GFP)-fusion protein localizes to the cytoplasm		
YMR247C	*RKR1*	RING domain E3 ubiquitin ligase; involved in ubiquitin-mediated degradation of non-stop proteins and translationally stalled ER membrane proteins; component of ribosome-bound RQC (ribosome quality control) complex; degrades products of mRNAs lacking a termination codon regardless of a poly(A) tail; functional connections to chromatin modification; homolog of mouse Listerin, mutations in which reported to cause neurodegeneration	*biological_process*	chromatin organization<br>ubiquitin-dependent protein catabolic process<br>proteasomal protein catabolic process<br>protein ubiquitination<br>protein metabolic process<br>protein catabolic process<br>subtelomeric heterochromatin formation<br>rescue of stalled ribosome<br>ribosome-associated ubiquitin-dependent protein catabolic process
			cellular_component	chromosome, telomeric region<br>nucleus<br>cytoplasm<br>cytosol<br>cytosolic ribosome<br>RQC complex
			molecular_function	ubiquitin-protein transferase activity<br>zinc ion binding<br>transferase activity<br>ribosomal large subunit binding<br>metal ion binding<br>ubiquitin protein ligase activity
YMR234W	*RNH1*	Ribonuclease H1; able to bind double-stranded RNAs and RNA-DNA hybrids; associates with RNAse polymerase I.	*biological_process*	RNA catabolic process<br>DNA replication, removal of RNA primer<br>nucleic acid metabolic process
			cellular_component	nucleus<br>cytoplasm
			molecular_function	magnesium ion binding<br>nucleic acid binding<br>nuclease activity<br>endonuclease activity<br>RNA-DNA hybrid ribonuclease activity<br>hydrolase activity<br>metal ion binding
YJL144W	*ROQ1*	Ub-ligase substrate-specificity factor; proteolytically-cleaved form acts as a	*biological_process*	cellular response to water deprivation<br>stress-induced homeostatically regulated protein degradation pathway
			cellular_component	cytoplasm
			molecular_function	ubiquitin-protein transferase regulator activity

		pseudosubstrate, binding and altering the substrate specificity of Ubr1p towards misfolded and native ER membrane and cytosolic proteins, as part of the stress-induced homeostatically regulated protein degradation (SHRED) pathway; hydrophilin essential during desiccation-rehydration; induced by osmotic stress, starvation and during stationary phase; protein abundance increases in response to DNA replication stress		
YMR214W	*SCJ1*	One of several homologs of bacterial chaperone DnaJ; located in the ER lumen where it cooperates with Kar2p to mediate maturation of proteins	*biological_process*	protein folding<br>response to unfolded protein<br>protein transport<br>protein folding in endoplasmic reticulum<br>ERAD pathway<br>protein refolding
			cellular_component	cytoplasm<br>endoplasmic reticulum<br>endoplasmic reticulum lumen
			molecular_function	zinc ion binding<br>Hsp70 protein binding<br>heat shock protein binding<br>metal ion binding<br>unfolded protein binding<br>protein-folding chaperone binding
YJL147C	*SMT1*	Translational repressor of the mitochondrial ATP6/8 mRNA; homozygous diploid deletion strain has a sporulation defect characterized by elevated dityrosine in the soluble fraction; expression	*biological_process*	biological_process
			cellular_component	mitochondrion
			molecular_function	molecular_function

		induced by calcium shortage		
YJL151C	*SNA3*	Protein involved in efficient MVB sorting of proteins to the vacuole; may function as an RSP5 adapter protein for MVB cargos; integral membrane protein localized to vacuolar intralumenal vesicles	*biological_process*	protein catabolic process<br>endosome transport via multivesicular body sorting pathway
			cellular_component	fungal-type vacuole<br>fungal-type vacuole lumen<br>endosome<br>vacuole<br>vacuolar lumen<br>membrane<br>cytoplasmic vesicle membrane<br>cytoplasmic vesicle<br>late endosome membrane
			molecular_function	ubiquitin protein ligase binding
YJR104C	*SOD1*	Cytosolic copper-zinc superoxide dismutase; also sulfide oxidase; detoxifies superoxide and hydrogen sulfide; stabilizes Yck1p and Yck2p kinases in glucose to repress respiration; phosphorylated by Dun1p, enters nucleus under oxidative stress to promote transcription of stress response genes; abundance increases under DNA replication stress; localization to mitochondrial intermembrane space is modulated by MICOS complex; human ortholog SOD1 implicated in ALS complements a null allele	*biological_process*	superoxide metabolic process<br>intracellular copper ion homeostasis<br>intracellular zinc ion homeostasis<br>removal of superoxide radicals<br>fungal-type cell wall organization<br>cellular response to oxidative stress<br>positive regulation of transcription by RNA polymerase II<br>protein stabilization<br>negative regulation of cellular respiration<br>cellular detoxification
			cellular_component	nucleus<br>cytoplasm<br>mitochondrion<br>mitochondrial intermembrane space<br>cytosol
			disease_ontology	amyotrophic lateral sclerosis
			molecular_function	superoxide dismutase activity<br>copper ion binding<br>zinc ion binding<br>antioxidant activity<br>oxidoreductase activity<br>oxidoreductase activity, acting on a sulfur group of donors, oxygen as acceptor<br>metal ion binding
YBR105C	*VID24*	GID Complex regulatory subunit; binds GID Complex in response to glucose through	*biological_process*	protein targeting to vacuole<br>protein catabolic process in the vacuole<br>protein transport<br>proteasome-mediated ubiquitin-dependent protein catabolic process

		interactions with complex member Vid28p; regulates fructose-1,6-bisphosphatase (FBPase) targeting to the vacuole; promotes proteasome-dependent catabolite degradation of FBPase; peripheral membrane protein located at Vid (vacuole import and degradation) vesicles		negative regulation of gluconeogenesis
			cellular_component	vacuole<br>cytosol<br>membrane<br>cytoplasmic vesicle membrane<br>cytoplasmic vesicle<br>GID complex
			molecular_function	molecular_function
YHR060W	*VMA22*	Protein that is required for vacuolar H+-ATPase (V-ATPase) function; peripheral membrane protein; not an actual component of the V-ATPase complex; functions in the assembly of the V-ATPase; localized to the yeast endoplasmic reticulum (ER)	*biological_process*	vacuolar acidification<br>vacuolar proton-transporting V-type ATPase complex assembly
			cellular_component	nucleus<br>Vma12-Vma22 assembly complex
			disease_ontology	congenital disorder of glycosylation type IIo
			molecular_function	unfolded protein binding

Table S4. Yeast GO Slim Mapper annotated terms of genes deleted in ZnS QD-sensitive mutants.

GO term	GO ID	Cluster frequency	Genome frequency	Annotated genes
**Molecular Function**				
ion binding	GO:0043167	13.33%	10.82%	YJR104C, YMR214W, YMR244C-A, YMR247C
oxidoreductase activity	GO:0016491	10.00%	4.64%	YJR104C, YMR170C, YPL091W
nuclease activity	GO:0004518	6.67%	2.16%	YMR234W, YMR285C
enzyme regulator activity	GO:0030234	6.67%	3.56%	YJL144W, YMR264W
unfolded protein binding	GO:0051082	6.67%	1.06%	YHR060W, YNL315C
transferase activity	GO:0016740	6.67%	1.20%	YMR307W, YPL148C
	other	26.64%		
kinase activity	GO:0016301	3.33%	3.07%	YPL188W
GTPase activity	GO:0003924	3.33%	0.99%	YMR173W
structural constituent of ribosome	GO:0003735	3.33%	3.55%	YNL122C
ATP hydrolysis activity	GO:0016887	3.33%	1.39%	YMR173W
ubiquitin-like protein binding	GO:0032182	3.33%	0.69%	YMR264W
enzyme binding	GO:0019899	3.33%	1.34%	YJL151C
ligase activity	GO:0016874	3.33%	1.77%	YMR246W
transmembrane transporter activity	GO:0022857	3.33%	5.30%	YOL103W
**Biological Process**				
response to chemical	GO:0042221	20.00%	6.32%	YJL144W, YJR104C, YMR214W, YMR264W, YPL091W, YPL188W
proteolysis involved in protein catabolic process	GO:0051603	16.67%	3.36%	YBR105C, YJL144W, YMR214W, YMR247C, YMR264W
intracellular monoatomic ion homeostasis	GO:0006873	10.00%	1.97%	YHR060W, YJR104C, YMR244C-A
response to oxidative stress	GO:0006979	6.67%	1.31%	YPL091W, YPL188W
transcription by RNA polymerase II	GO:0006366	6.67%	7.54%	YJR104C, YMR164C
mitochondrion organization	GO:0007005	6.67%	3.86%	YMR244C-A, YNL315C
cell wall organization or biogenesis	GO:0071554	6.67%	3.02%	YJR104C, YMR307W
lipid metabolic process	GO:0006629	6.67%	4.64%	YMR160W, YMR246W
chromatin organization	GO:0006325	6.67%	3.84%	YMR247C, YMR307W
DNA repair	GO:0006281	6.67%	4.12%	YJL142C, YMR173W
nucleobase-containing small molecule metabolic process	GO:0055086	6.67%	2.87%	YMR246W, YPL188W
lipid transport	GO:0006869	6.67%	1.11%	YMR162C, YMR246W
	other	66.60%		
cellular respiration	GO:0045333	3.33%	1.31%	YJR104C

DNA recombination	GO:0006310	3.33%	2.96%	YJL142C
protein modification by small protein conjugation or removal	GO:0070647	3.33%	2.19%	YMR247C
nucleobase-containing compound transport	GO:0015931	3.33%	2.14%	YMR255W
amino acid metabolic process	GO:0006520	3.33%	2.44%	YMR170C
organelle fusion	GO:0048284	3.33%	1.53%	YMR232W
telomere organization	GO:0032200	3.33%	2.07%	YMR247C
pseudohyphal growth	GO:0007124	3.33%	1.13%	YMR162C
regulation of transport	GO:0051049	3.33%	1.33%	YPR115W
transmembrane transport	GO:0055085	3.33%	4.16%	YPR115W
carbohydrate transport	GO:0008643	3.33%	0.56%	YPR115W
carbohydrate metabolic process	GO:0005975	3.33%	2.27%	YBR105C
translational elongation	GO:0006414	3.33%	5.15%	YMR247C
endosomal transport	GO:0016197	3.33%	1.33%	YJL151C
protein folding	GO:0006457	3.33%	1.51%	YMR214W
protein targeting	GO:0006605	3.33%	3.33%	YBR105C
RNA catabolic process	GO:0006401	3.33%	2.45%	YMR234W
rRNA processing	GO:0006364	3.33%	5.10%	YMR285C
mitochondrial translation	GO:0032543	3.33%	2.62%	YNL122C
nucleus organization	GO:0006997	3.33%	1.51%	YMR232W

Cellular Component

mitochondrion	GO:0005739	40.00%	17.61%	YDR033W, YJL147C, YJR104C, YMR232W, YMR244C-A, YMR264W, YMR307W, YNL122C, YNL315C, YPL091W, YPL148C, YPL188W
cytoplasm	GO:0005737	36.67%	24.38%	YJL144W, YMR170C, YMR173W, YMR232W, YMR234W, YMR244C-A, YMR246W, YMR255W, YMR285C, YPL091W, YPR115W
nucleus	GO:0005634	30.00%	23.63%	YHR060W, YJR104C, YMR164C, YMR232W, YMR234W, YMR244C-A, YMR247C, YMR307W, YPL091W
plasma membrane	GO:0005886	16.67%	5.85%	YDR033W, YMR162C, YMR246W, YMR307W, YOL103W
endoplasmic reticulum	GO:0005783	13.33%	9.44%	YDR033W, YMR162C, YMR214W, YMR264W
membrane	GO:0016020	13.33%	13.11%	YJL151C, YMR160W, YMR264W, YMR307W

cytoplasmic vesicle	GO:0031410	10.00%	1.22%	YBR105C, YMR162C, YMR307W
vacuole	GO:0005773	10.00%	7.33%	YJL151C, YMR160W, YOL103W
mitochondrial envelope	GO:0005740	6.67%	4.47%	YJR104C, YMR244C-A
peroxisome	GO:0005777	6.67%	2.07%	YMR160W, YPL091W
site of polarized growth	GO:0030427	6.67%	4.19%	YMR162C, YMR232W
	other	9.99%		
Golgi apparatus	GO:0005794	3.33%	2.59%	YMR162C
cell wall	GO:0005618	3.33%	1.54%	YMR307W
endomembrane system	GO:0012505	3.33%	0.43%	YMR162C

Table S5. Percentage value of detected events in each dot plot quadrant in flow cytometry analyses**.** Q1, upper left quadrant; Q2, upper right quadrant; Q3, lower left quadrant; Q4, lower right quadrant.

	wild-type				*pos5Δ*			
PI signal (dead cells)	+	+	-	-	+	+	-	-
DCF signal (oxidative stress)	-	+	-	+	-	+	-	+
	Q1	Q2	Q3	Q4	Q1	Q2	Q3	Q4
untreated	0.42%	0.21%	99.25%	0.12%	1.05%	0.02%	98.85%	0.08%
100 mg L^{-1} ZnS QDs	0.42%	0.20%	99.11%	0.27%	4.11%	0.20%	95.24%	0.44%
H_2O_2 (0.3%)	0.23%	0.43%	74.31%	25.03%	1.91%	0.56%	94.70%	2.83%
H_2O_2 (1.5%)	0.81%	1.31%	61.32%	36.56%	2.63%	0.80%	89.06%	7.50%
H_2O_2 (3%)	5.66%	3.61%	46.37%	44.36%	9.94%	6.31%	65.45%	18.30%
95 °C	81.63%	18.02%	0.15%	0.20%	97.44%	2.36%	0.17%	0.03%

Table S6. Enriched annotation terms from Functional Annotation Chart (David) in each tested condition. (Pvalue < 0.05; Benjamini < 0.05; 5-hour treatment, early response; 24-hour treatment, late response).

pos5Δ, untreated

Category	GO Term	P-Value	Regulation
early response			
UP_KW_BIOLOGICAL_PROCESS	Ribosome biogenesis	8.60E-06	Down-regulated
GOTERM_CC_DIRECT	nucleolus	1.70E-05	Down-regulated
GOTERM_CC_DIRECT	fungal-type cell wall	1.50E-04	Down-regulated
GOTERM_MF_DIRECT	acid phosphatase activity	1.70E-04	Down-regulated
GOTERM_CC_DIRECT	preribosome, large subunit precursor	1.30E-03	Down-regulated
GOTERM_BP_DIRECT	sulfate assimilation	1.60E-06	Up-regulated
GOTERM_CC_DIRECT	membrane	3.40E-05	Up-regulated
UP_KW_BIOLOGICAL_PROCESS	Amino-acid biosynthesis	3.70E-05	Up-regulated
UP_KW_MOLECULAR_FUNCTION	Chaperone	3.80E-04	Up-regulated
UP_KW_MOLECULAR_FUNCTION	Oxidoreductase	4.10E-04	Up-regulated
UP_KW_BIOLOGICAL_PROCESS	Methionine biosynthesis	1.00E-03	Up-regulated
UP_KW_BIOLOGICAL_PROCESS	Transport	1.50E-03	Up-regulated
late response			
GOTERM_CC_DIRECT	cytosolic large ribosomal subunit	5.50E-07	Down-regulated
GOTERM_BP_DIRECT	translation	1.30E-06	Down-regulated
GOTERM_CC_DIRECT	cytoplasm	3.30E-06	Down-regulated

GOTERM_BP_DIRECT	cytoplasmic translation	4.60E-06	Down-regulated
GOTERM_BP_DIRECT	telomere maintenance via recombination	1.00E-05	Down-regulated
GOTERM_MF_DIRECT	DNA helicase activity	1.40E-05	Down-regulated
GOTERM_CC_DIRECT	ribonucleoprotein complex	2.70E-05	Down-regulated
UP_KW_CELLULAR_COMPONENT	Secreted	6.10E-05	Down-regulated
GOTERM_CC_DIRECT	ribosome	8.20E-05	Down-regulated
GOTERM_CC_DIRECT	extracellular region	1.10E-04	Down-regulated
UP_KW_CELLULAR_COMPONENT	Cell wall	1.90E-04	Down-regulated
GOTERM_CC_DIRECT	mitochondrion	5.00E-06	Up-regulated
GOTERM_MF_DIRECT	oxidoreductase activity	6.30E-06	Up-regulated
UP_KW_MOLECULAR_FUNCTION	Oxidoreductase	1.20E-05	Up-regulated
UP_KW_MOLECULAR_FUNCTION	Chaperone	1.40E-05	Up-regulated
GOTERM_MF_DIRECT	antioxidant activity	9.20E-05	Up-regulated
GOTERM_MF_DIRECT	copper ion binding	2.70E-04	Up-regulated
GOTERM_CC_DIRECT	cytoplasm	3.30E-04	Up-regulated

pos5Δ, $ZnSO_4$

**Category**	**GO Term**	**P-Value**	**Regulation**
early response			
GOTERM_CC_DIRECT	ribonucleoprotein complex	3.20E-14	Up-regulated
GOTERM_MF_DIRECT	structural constituent of ribosome	4.00E-14	Up-regulated
GOTERM_CC_DIRECT	ribosome	5.40E-14	Up-regulated

UP_KW_MOLECULAR_FUNCTION	Ribonucleoprotein	1.80E-13	Up-regulated
GOTERM_CC_DIRECT	cytosolic small ribosomal subunit	1.40E-09	Up-regulated
GOTERM_BP_DIRECT	translation	4.60E-09	Up-regulated
GOTERM_BP_DIRECT	cytoplasmic translation	1.90E-08	Up-regulated
GOTERM_CC_DIRECT	small ribosomal subunit	4.30E-04	Up-regulated
GOTERM_CC_DIRECT	ribosomal subunit	4.60E-04	Up-regulated
GOTERM_CC_DIRECT	cytoplasm	5.50E-04	Up-regulated
GOTERM_CC_DIRECT	precatalytic spliceosome	1.50E-03	Up-regulated
UP_KW_CELLULAR_COMPONENT	Cytoplasm	2.70E-03	Up-regulated
GOTERM_CC_DIRECT	U2 snRNP	2.90E-03	Up-regulated
GOTERM_CC_DIRECT	pICln-Sm protein complex	3.10E-03	Up-regulated
late response			
GOTERM_CC_DIRECT	nucleus	3.60E-08	Down-regulated
GOTERM_BP_DIRECT	rRNA processing	2.30E-07	Down-regulated
GOTERM_CC_DIRECT	ribonucleoprotein complex	1.20E-05	Down-regulated
UP_KW_BIOLOGICAL_PROCESS	rRNA processing	2.50E-05	Down-regulated
UP_KW_MOLECULAR_FUNCTION	Ribonucleoprotein	9.70E-05	Down-regulated
GOTERM_CC_DIRECT	mitochondrial large ribosomal subunit	1.50E-04	Down-regulated
GOTERM_CC_DIRECT	nucleolus	6.70E-04	Down-regulated
UP_KW_BIOLOGICAL_PROCESS	Transcription regulation	8.60E-04	Down-regulated
UP_KW_CELLULAR_COMPONENT	Cell wall	2.80E-05	Up-regulated

GOTERM_CC_DIRECT	extracellular region	4.20E-05	Up-regulated
UP_KW_CELLULAR_COMPONENT	Secreted	4.50E-05	Up-regulated

pos5Δ, ZnS QDs

**Category**	**GO Term**	**P-Value**	**Regulation**
early response			
UP_KW_CELLULAR_COMPONENT	Cell wall	7.10E-05	Down-regulated
GOTERM_CC_DIRECT	fungal-type cell wall	1.80E-04	Down-regulated
UP_KW_CELLULAR_COMPONENT	Secreted	4.10E-04	Down-regulated
GOTERM_MF_DIRECT	structural constituent of ribosome	1.10E-17	Up-regulated
UP_KW_MOLECULAR_FUNCTION	Ribosomal protein	3.10E-17	Up-regulated
GOTERM_CC_DIRECT	ribosome	8.70E-16	Up-regulated
UP_KW_MOLECULAR_FUNCTION	Ribonucleoprotein	1.00E-15	Up-regulated
GOTERM_CC_DIRECT	ribonucleoprotein complex	4.40E-14	Up-regulated
GOTERM_BP_DIRECT	cytoplasmic translation	4.60E-12	Up-regulated
GOTERM_BP_DIRECT	translation	1.10E-11	Up-regulated
GOTERM_CC_DIRECT	cytosolic small ribosomal subunit	1.80E-09	Up-regulated
UP_KW_BIOLOGICAL_PROCESS	Protein transport	1.00E-04	Up-regulated
UP_KW_CELLULAR_COMPONENT	Mitochondrion	1.10E-04	Up-regulated
GOTERM_CC_DIRECT	cytoplasm	1.30E-04	Up-regulated
GOTERM_CC_DIRECT	cytosolic large ribosomal subunit	4.20E-04	Up-regulated
GOTERM_CC_DIRECT	small ribosomal subunit	5.80E-04	Up-regulated

UP_KW_BIOLOGICAL_PROCESS	Electron transport	1.30E-03	Up-regulated
GOTERM_CC_DIRECT	mitochondrial intermembrane space	1.30E-03	Up-regulated
UP_KW_CELLULAR_COMPONENT	Cytoplasm	1.80E-03	Up-regulated
UP_KW_BIOLOGICAL_PROCESS	Transport	2.20E-03	Up-regulated
late response			
UP_KW_MOLECULAR_FUNCTION	Ribosomal protein	1.70E-03	Up-regulated
UP_KW_MOLECULAR_FUNCTION	Ribonucleoprotein	2.10E-03	Up-regulated

wild-type, $ZnSO_4$

**Category**	**GO Term**	**P-Value**	**Regulation**
early response			
UP_KW_MOLECULAR_FUNCTION	Chaperone	8.40E-08	Up-regulated
GOTERM_CC_DIRECT	integral component of Golgi membrane	1.00E-06	Up-regulated
GOTERM_CC_DIRECT	endoplasmic reticulum membrane	4.10E-06	Up-regulated
GOTERM_CC_DIRECT	membrane	4.50E-06	Up-regulated
GOTERM_CC_DIRECT	Golgi membrane	5.10E-06	Up-regulated
GOTERM_MF_DIRECT	chaperone binding	2.50E-05	Up-regulated
UP_KW_CELLULAR_COMPONENT	Endoplasmic reticulum	4.10E-05	Up-regulated
UP_KW_BIOLOGICAL_PROCESS	Transport	3.60E-04	Up-regulated
GOTERM_CC_DIRECT	Golgi apparatus	5.20E-04	Up-regulated
UP_KW_CELLULAR_COMPONENT	Golgi apparatus	2.20E-03	Up-regulated
late response			
GOTERM_CC_DIRECT	nucleus	3.10E-14	Down-regulated

GOTERM_BP_DIRECT	positive regulation of transcription from RNA polymerase II promoter	3.70E-07	Down-regulated
GOTERM_BP_DIRECT	chromosome segregation	5.60E-06	Down-regulated
GOTERM_CC_DIRECT	small-subunit processome	6.80E-06	Down-regulated
GOTERM_BP_DIRECT	ribosome biogenesis	1.10E-05	Down-regulated
GOTERM_CC_DIRECT	nucleoplasm	2.20E-05	Down-regulated
UP_KW_BIOLOGICAL_PROCESS	Transcription	4.40E-05	Down-regulated
GOTERM_BP_DIRECT	protein phosphorylation	7.00E-05	Down-regulated
UP_KW_BIOLOGICAL_PROCESS	Transcription regulation	8.30E-05	Down-regulated
GOTERM_BP_DIRECT	chromatin remodeling	9.20E-05	Down-regulated
GOTERM_BP_DIRECT	regulation of transcription, DNA-templated	9.30E-05	Down-regulated
GOTERM_CC_DIRECT	nucleolus	1.30E-04	Down-regulated
GOTERM_CC_DIRECT	cytoplasm	1.80E-04	Down-regulated
GOTERM_BP_DIRECT	mitotic spindle assembly checkpoint	2.30E-04	Down-regulated

GOTERM_BP_DIRECT	maturation of SSU-rRNA from tricistronic rRNA transcript (SSU-rRNA, 5.8S rRNA, LSU-rRNA)	2.50E-04	Down-regulated
UP_KW_MOLECULAR_FUNCTION	Activator	6.50E-04	Down-regulated
UP_KW_CELLULAR_COMPONENT	Mitochondrion	7.10E-17	Up-regulated
GOTERM_CC_DIRECT	mitochondrial inner membrane	2.50E-12	Up-regulated
GOTERM_BP_DIRECT	cellular respiration	4.40E-11	Up-regulated
UP_KW_BIOLOGICAL_PROCESS	Stress response	8.90E-11	Up-regulated
UP_KW_CELLULAR_COMPONENT	Mitochondrion inner membrane	8.60E-10	Up-regulated
GOTERM_BP_DIRECT	protein folding	9.20E-10	Up-regulated
UP_KW_MOLECULAR_FUNCTION	Chaperone	1.40E-08	Up-regulated
GOTERM_MF_DIRECT	unfolded protein binding	1.50E-08	Up-regulated
UP_KW_BIOLOGICAL_PROCESS	ATP synthesis	1.90E-08	Up-regulated
GOTERM_MF_DIRECT	proton-transporting ATP synthase activity, rotational mechanism	1.90E-08	Up-regulated
GOTERM_CC_DIRECT	mitochondrial intermembrane space	4.30E-08	Up-regulated

GOTERM_BP_DIRECT	ATP synthesis coupled proton transport	5.60E-08	Up-regulated
GOTERM_BP_DIRECT	ATP biosynthetic process	5.80E-08	Up-regulated
GOTERM_BP_DIRECT	hydrogen ion transmembrane transport	6.80E-08	Up-regulated
GOTERM_BP_DIRECT	protein refolding	2.80E-07	Up-regulated
GOTERM_BP_DIRECT	cellular response to heat	3.30E-07	Up-regulated
GOTERM_BP_DIRECT	oxidative phosphorylation	4.20E-07	Up-regulated
GOTERM_MF_DIRECT	chaperone binding	1.10E-06	Up-regulated
GOTERM_CC_DIRECT	mitochondrial respiratory chain complex IV	3.20E-06	Up-regulated
GOTERM_MF_DIRECT	antioxidant activity	3.90E-06	Up-regulated
GOTERM_BP_DIRECT	mitochondrial electron transport, cytochrome c to oxygen	6.50E-06	Up-regulated
UP_KW_BIOLOGICAL_PROCESS	Electron transport	9.00E-06	Up-regulated
GOTERM_BP_DIRECT	tricarboxylic acid cycle	1.30E-05	Up-regulated
UP_KW_BIOLOGICAL_PROCESS	Hydrogen ion transport	1.90E-05	Up-regulated

GOTERM_CC_DIRECT	proton-transporting ATP synthase complex, coupling factor F(o)	2.00E-05	Up-regulated
GOTERM_MF_DIRECT	cytochrome-c oxidase activity	2.20E-05	Up-regulated
UP_KW_CELLULAR_COMPONENT	CF(0)	2.70E-05	Up-regulated
GOTERM_CC_DIRECT	mitochondrial membrane	2.80E-05	Up-regulated
GOTERM_CC_DIRECT	mitochondrial proton-transporting ATP synthase complex, coupling factor F(o)	3.80E-05	Up-regulated
UP_KW_MOLECULAR_FUNCTION	Oxidoreductase	8.60E-05	Up-regulated
GOTERM_MF_DIRECT	oxidoreductase activity	1.00E-04	Up-regulated
GOTERM_BP_DIRECT	chaperone mediated protein folding requiring cofactor	1.10E-04	Up-regulated
GOTERM_BP_DIRECT	protein stabilization	2.20E-04	Up-regulated
GOTERM_BP_DIRECT	aerobic respiration	2.50E-04	Up-regulated
UP_KW_CELLULAR_COMPONENT	Cell wall	3.40E-04	Up-regulated
UP_KW_MOLECULAR_FUNCTION	Antioxidant	4.10E-04	Up-regulated
GOTERM_MF_DIRECT	glutathione peroxidase activity	4.30E-04	Up-regulated

GOTERM_BP_DIRECT	cellular oxidant detoxification	5.60E-04	Up-regulated
GOTERM_BP_DIRECT	mitochondrial respiratory chain complex IV assembly	5.60E-04	Up-regulated
GOTERM_MF_DIRECT	heat shock protein binding	7.10E-04	Up-regulated
GOTERM_CC_DIRECT	extracellular region	7.90E-04	Up-regulated
GOTERM_BP_DIRECT	cellular response to oxidative stress	8.20E-04	Up-regulated
GOTERM_CC_DIRECT	cytoplasm	8.30E-04	Up-regulated
UP_KW_BIOLOGICAL_PROCESS	Ion transport	8.40E-04	Up-regulated
UP_KW_CELLULAR_COMPONENT	Secreted	8.60E-04	Up-regulated
GOTERM_BP_DIRECT	mitochondrial ATP synthesis coupled proton transport	9.40E-04	Up-regulated
UP_KW_MOLECULAR_FUNCTION	Ribosomal protein	1.20E-03	Up-regulated
UP_KW_MOLECULAR_FUNCTION	Peroxidase	4.30E-03	Up-regulated

wild-type, ZnSQDs

**Category**	**GO Term**	**P-Value**	**Regulation**
early response			
GOTERM_CC_DIRECT	endoplasmic reticulum membrane	4.40E-10	Up-regulated
GOTERM_CC_DIRECT	membrane	7.20E-08	Up-regulated
UP_KW_CELLULAR_COMPONENT	Endoplasmic reticulum	2.20E-07	Up-regulated

GOTERM_CC_DIRECT	ER to Golgi transport vesicle	3.90E-05	Up-regulated
GOTERM_CC_DIRECT	integral component of membrane	9.50E-05	Up-regulated
GOTERM_CC_DIRECT	Golgi membrane	1.80E-04	Up-regulated
GOTERM_CC_DIRECT	integral component of Golgi membrane	3.00E-04	Up-regulated
UP_KW_BIOLOGICAL_PROCESS	Transport	4.20E-04	Up-regulated
UP_KW_MOLECULAR_FUNCTION	Chaperone	5.20E-04	Up-regulated
UP_KW_BIOLOGICAL_PROCESS	Protein transport	7.30E-04	Up-regulated
GOTERM_CC_DIRECT	Golgi apparatus	1.90E-03	Up-regulated
late response			
GOTERM_BP_DIRECT	chromatin remodeling	6.50E-07	Down-regulated
GOTERM_CC_DIRECT	nucleus	7.00E-06	Down-regulated
GOTERM_BP_DIRECT	chromatin organization	1.70E-05	Down-regulated
UP_KW_MOLECULAR_FUNCTION	Chromatin regulator	4.40E-05	Down-regulated
UP_KW_MOLECULAR_FUNCTION	Ribosomal protein	5.00E-12	Up-regulated
GOTERM_MF_DIRECT	structural constituent of ribosome	6.80E-12	Up-regulated
GOTERM_CC_DIRECT	ribosome	5.90E-11	Up-regulated
UP_KW_MOLECULAR_FUNCTION	Ribonucleoprotein	2.70E-09	Up-regulated
GOTERM_CC_DIRECT	ribonucleoprotein complex	2.70E-08	Up-regulated
UP_KW_CELLULAR_COMPONENT	Mitochondrion	4.80E-07	Up-regulated
GOTERM_BP_DIRECT	cytoplasmic translation	6.30E-07	Up-regulated

GOTERM_BP_DIRECT	translation	1.60E-06	Up-regulated
GOTERM_CC_DIRECT	mitochondrial respiratory chain complex IV	2.20E-06	Up-regulated
GOTERM_BP_DIRECT	mitochondrial electron transport, cytochrome c to oxygen	3.80E-06	Up-regulated
UP_KW_CELLULAR_COMPONENT	Mitochondrion inner membrane	1.30E-05	Up-regulated
GOTERM_BP_DIRECT	cellular respiration	2.00E-05	Up-regulated
GOTERM_CC_DIRECT	cytosolic small ribosomal subunit	2.50E-05	Up-regulated
GOTERM_BP_DIRECT	hydrogen ion transmembrane transport	5.50E-05	Up-regulated
GOTERM_CC_DIRECT	mitochondrial inner membrane	6.40E-05	Up-regulated
GOTERM_BP_DIRECT	oxidative phosphorylation	7.90E-05	Up-regulated
GOTERM_CC_DIRECT	mitochondrial intermembrane space	1.10E-04	Up-regulated
GOTERM_BP_DIRECT	mitochondrial respiratory chain complex IV assembly	1.10E-04	Up-regulated
GOTERM_MF_DIRECT	cytochrome-c oxidase activity	5.00E-04	Up-regulated

Table S7. List of DEGs associated with Cellular Component "Mitochondrion" modulated across treatments for wild-type and *pos5Δ*.

Wild type

Standard Name	Systematic Name	Name
ABF2	YMR072W	ARS-Binding Factor
ACO1	YLR304C	ACOnitase
ADK1	YDR226W	ADenylate Kinase
AIM19	YIL087C	Altered Inheritance rate of Mitochondria
AIM41	YOR215C	Altered Inheritance of Mitochondria
ATP10	YLR393W	ATP synthase
ATP14	YLR295C	ATP synthase
ATP15	YPL271W	ATP synthase
ATP16	YDL004W	ATP synthase
ATP17	YDR377W	ATP synthase
ATP18	YML081C-A	ATP synthase
ATP19	YOL077W-A	ATP synthase
ATP20	YPR020W	ATP synthase
ATP3	YBR039W	ATP synthase
ATP4	YPL078C	ATP synthase
ATP5	YDR298C	ATP synthase
ATP7	YKL016C	ATP synthase
CHA1	YCL064C	Catabolism of Hydroxy Amino acids
CIS1	YLR346C	CItrinin Sensitive knockout
CIT1	YNR001C	CITrate synthase
CMC1	YKL137W	Cx9C Mitochondrial protein necessary for full assembly of Cytochrome c oxidase
CMC2	YBL059C-A	Cx9C Motif-Containing protein
COA2	YPL189C-A	Cytochrome Oxidase Assembly
COA3	YJL062W-A	Cytochrome Oxidase Assembly
COA4	YLR218C	Cytochrome Oxidase Assembly factor
COA6	YMR244C-A	Cytochrome Oxidase Assembly
COX12	YLR038C	Cytochrome c OXidase
COX13	YGL191W	Cytochrome c OXidase
COX17	YLL009C	Cytochrome c OXidase
COX26	YDR119W-A	-
COX4	YGL187C	Cytochrome c OXidase
COX5A	YNL052W	Cytochrome c OXidase
COX6	YHR051W	Cytochrome c OXidase
COX7	YMR256C	Cytochrome c OXidase
COX8	YLR395C	Cytochrome c OXidase
COX9	YDL067C	Cytochrome c OXidase
CPR3	YML078W	Cyclosporin A-sensitive Proline Rotamase
CYC1	YJR048W	CYtochrome C
FMC1	YIL098C	Formation of Mitochondrial Complexes
FMP16	YDR070C	Found in Mitochondrial Proteome
FMP33	YJL161W	Found in Mitochondrial Proteome
FMP46	YKR049C	Found in Mitochondrial Proteome
FUM1	YPL262W	FUMarase

FYV4	YHR059W	Function required for Yeast Viability
GEP3	YOR205C	GEnetic interactors of Prohibitins
GEP5	YLR091W	GEnetic interactors of Prohibitins
GPX1	YKL026C	Glutathione PeroXidase
GRX2	YDR513W	GlutaRedoXin
HMF1	YER057C	Homologous Mmf1p Factor
HSC82	YMR186W	-
HSP10	YOR020C	Heat Shock Protein
HSP60	YLR259C	Heat Shock Protein
HSP78	YDR258C	Heat Shock Protein
HYR1	YIR037W	HYdroperoxide Resistance
IMG1	YCR046C	Integrity of Mitochondrial Genome
IMG2	YCR071C	Integrity of Mitochondrial Genome
JAC1	YGL018C	J-type Accessory Chaperone
MCO6	YJL127C-B	Mitochondrial Class One protein of 6 kDa
MCR1	YKL150W	Mitochondrial NADH-Cytochrome b5 Reductase
MDH1	YKL085W	Malate DeHydrogenase
MIC27	YNL100W	MItochondrial contact site and Cristae organizing system
MIX14	YDR031W	Mitochondrial Intermembrane space CX(n)C motif protein
MRH1	YDR033W	Membrane protein Related to Hsp30p
MRM2	YGL136C	Mitochondrial rRNA Methyl transferase
MRP10	YDL045W-A	Mitochondrial Ribosomal Protein
MRP17	YKL003C	Mitochondrial Ribosomal Protein
MRP21	YBL090W	Mitochondrial Ribosomal Protein
MRPL19	YNL185C	Mitochondrial Ribosomal Protein, Large subunit
MRPL23	YOR150W	Mitochondrial Ribosomal Protein, Large subunit
MRPL33	YMR286W	Mitochondrial Ribosomal Protein, Large subunit
MRPL51	YPR100W	Mitochondrial Ribosomal Protein, Large subunit
MRPL6	YHR147C	Mitochondrial Ribosomal Protein, Large subunit
MRPL9	YGR220C	Mitochondrial Ribosomal Protein, Large subunit
MRPS12	YNR036C	Mitochondrial Ribosomal Protein, Small subunit
MRPS8	YMR158W	Mitochondrial Ribosomal Protein, Small subunit
MTC3	YGL226W	Maintenance of Telomere Capping
NCA3	YJL116C	Nuclear Control of ATPase
NIF3	YGL221C	Ngg1p-Interacting Factor
NTG2	YOL043C	eNdonuclease Three-like Glycosylase
OM45	YIL136W	Outer Membrane
OPI3	YJR073C	OverProducer of Inositol
OXR1	YPL196W	OXidation Resistance
PAM16	YJL104W	Presequence translocase-Associated Motor
PAM17	YKR065C	Presequence translocase-Associated Motor
PAM18	YLR008C	Presequence translocase-Associated Motor
PET117	YER058W	PETite colonies
PET191	YJR034W	PETite colonies
PET9	YBL030C	PETite
POR1	YNL055C	PORin
PUS5	YLR165C	PseudoUridine Synthase
PUT1	YLR142W	Proline UTilization
QCR10	YHR001W-A	ubiQuinol-cytochrome C oxidoReductase
QCR6	YFR033C	ubiQuinol-cytochrome C oxidoReductase
QCR7	YDR529C	ubiQuinol-cytochrome C oxidoReductase

QCR8	YJL166W	ubiQuinol-cytochrome C oxidoReductase
RCF2	YNR018W	Respiratory superComplex Factor
RSM19	YNR037C	Ribosomal Small subunit of Mitochondria
RTC6	YPL183W-A	Restriction of Telomere Capping
SDH2	YLL041C	Succinate DeHydrogenase
SDH3	YKL141W	Succinate DeHydrogenase
SDH4	YDR178W	Succinate DeHydrogenase
SDH5	YOL071W	Succinate DeHydrogenase
SDH6	YDR379C-A	-
SDH8	YBR269C	Succinate DeHydrogenase
SEN2	YLR105C	Splicing ENdonuclease
SHH3	YMR118C	SDH3 Homolog
SOD1	YJR104C	SuperOxide Dismutase
SOM1	YEL059C-A	SOrting Mitochondrial
STF1	YDL130W-A	STabilizing Factor
STF2	YGR008C	STabilizing Factor
STP4	YDL048C	protein with similarity to Stp1p
TAR1	YLR154W-C	Transcript Antisense to Ribosomal RNA
TDH3	YGR192C	Triose-phosphate DeHydrogenase
TIM13	YGR181W	Translocase of the Inner Mitochondrial membrane
TIM17	YJL143W	Translocase of the Inner Mitochondrial membrane
TIM9	YEL020W-A	Translocase of the Inner Mitochondrial membrane
TMH11	YJR085C	TMem14 Homolog of 11 kDa
TMH18	YPR098C	TMem205 Homolog of 18 kDa
TOM6	YOR045W	Translocase of the Outer Mitochondrial membrane
TRX3	YCR083W	ThioRedoXin
WWM1	YFL010C	WW domain containing protein interacting with Metacaspase
XDJ1	YLR090W	-
YHM2	YMR241W	Yeast suppressor of HM mutant
YIA6	YIL006W	-

pos5Δ

**Standard Name**	**Systematic Name**	**Name**
ATG44	YIL156W-B	AuTophaGy related
ATP19	YOL077W-A	ATP synthase
CAB5	YDR196C	-
CMC1	YKL137W	Cx9C Mitochondrial protein necessary for full assembly of Cytochrome c oxidase
CMC2	YBL059C-A	Cx9C Motif-Containing protein
COI1	YDR381C-A	Cytochrome c Oxidase Interacting protein
COX13	YGL191W	Cytochrome c OXidase
COX17	YLL009C	Cytochrome c OXidase
COX7	YMR256C	Cytochrome c OXidase
CYC7	YEL039C	CYtochrome C
FIS1	YIL065C	mitochondrial FISsion
FMP46	YKR049C	Found in Mitochondrial Proteome
GPX2	YBR244W	Glutathione PeroXidase
HOT13	YKL084W	Helper Of Tim
IMP2	YMR035W	Inner Membrane Protease
INA17	YPL099C	INner membrane Assembly 17 kDa

MCO10	YOR020W-A	Mitochondrial Class One protein of 10 kDa
MCO6	YJL127C-B	Mitochondrial Class One protein of 6 kDa
MDM35	YKL053C-A	Mitochondrial Distribution and Morphology
MIM1	YOL026C	Mitochondrial IMport
MIX14	YDR031W	Mitochondrial Intermembrane space CX(n)C motif protein
MRP17	YKL003C	Mitochondrial Ribosomal Protein
MRP2	YPR166C	Mitochondrial Ribosomal Protein
MRP21	YBL090W	Mitochondrial Ribosomal Protein
MRPL20	YKR085C	Mitochondrial Ribosomal Protein, Large subunit
MRPL32	YCR003W	Mitochondrial Ribosomal Protein, Large subunit
MRPL49	YJL096W	Mitochondrial Ribosomal Protein, Large subunit
MRPL51	YPR100W	Mitochondrial Ribosomal Protein, Large subunit
MRPL6	YHR147C	Mitochondrial Ribosomal Protein, Large subunit
MRX14	YDR115W	Mitochondrial oRganization of gene eXpression (MIOREX)
QCR7	YDR529C	ubiQuinol-cytochrome C oxidoReductase
QCR8	YJL166W	ubiQuinol-cytochrome C oxidoReductase
QCR9	YGR183C	ubiQuinol-cytochrome C oxidoReductase
RDL1	YOR285W	RhoDanese-Like protein
RSM27	YGR215W	Ribosomal Small subunit of Mitochondria
SDH6	YDR379C-A	-
STF2	YGR008C	STabilizing Factor
SYM1	YLR251W	Stress-inducible Yeast Mpv17
TIM11	YDR322C-A	Translocase of the Inner Mitochondrial membrane
TIM8	YJR135W-A	Translocase of the Inner Mitochondrial membrane
TIM9	YEL020W-A	Translocase of the Inner Mitochondrial membrane
TMA19	YKL056C	Translation Machinery Associated
TMH11	YJR085C	TMem14 Homolog of 11 kDa
TOM7	YNL070W	Translocase of the Outer Mitochondrial membrane
TRX1	YLR043C	ThioRedoXin

Table S8. List of genes identified by the comparison of ZnS QDs and CdS QDs chemogenomic and transcriptomic data.

Systematic Name	Standard Name	Name	Homologue Standard Name	Condition
YAL054C	*ACS1*	Acetyl CoA Synthetase	*ACSS1; ACSS2*	down-regulated
YDR384C	*ATO3*	Ammonia (Ammonium) Transport Outward	*ND*	down-regulated
YKL190W	*CNB1*	CalciNeurin subunit B	*PPP3R1; PPP3R2*	up-regulated
YLL009C	*COX17*	Cytochrome c OXidase	*COX17*	up-regulated
YLR307C-A	*DPA10*	Delta-Psi dependent mitochondrial Assembly protein of 10 kDa	*ND*	down-regulated
YDR038C	*ENA5*	Exitus NAtru (Latin, "exit sodium")	*ND*	down-regulated
YMR020W	*FMS1*	Fenpropimorph-resistance Multicopy Suppressor	*KDM1B; SMOX*	down-regulated
YOL123W	*HRP1*	Heterogenous nuclear RibonucleoProtein	*HNRNPAB; HNRNPA1; HNRNPA3; HNRNPD; HNRNPDL; MSI1*	down-regulated
YER065C	*ICL1*	IsoCitrate Lyase	*ND*	down-regulated
YBR297W	*MAL33*	MALtose	*ND*	down-regulated
YPL187W	*MF(ALPHA)1*	Mating Factor ALPHA	*ND*	up-regulated
YGL226W	*MTC3*	Maintenance of Telomere Capping	*ND*	up-regulated
YMR170C	*ALD2*	ALdehyde Dehydrogenase	*ALDH1A1; ALDH1A2; ALDH1A3; ALDH1B1; ALDH1L1; ALDH2*	deletion mutant
YMR247C	*RKR1*	RING domain mutant Killed by Rtf1 deletion	*LTN1*	deletion mutant
YMR214W	*SCJ1*	S. Cerevisiae DnaJ	*DNAJB11*	deletion mutant
YMR246W	*CUE1*	Coupling of Ubiquitin conjugation to ER degradation	*ACSL3; ACSL4*	deletion mutant
YMR264W	*FAA4*	Fatty Acid Activation	*ND*	deletion mutant
YNL195C	*ND*	ND	*ND*	down-regulated
YNL040W	*ND*	ND	*PTGES3L-AARSD1; AARSD1*	down-regulated
YGR067C	*ND*	ND	*ND*	down-regulated
YER053C-A	*ND*	ND	*ND*	down-regulated
YPR168W	*NUT2*	Negative regulation of URS Two	*MED10*	down-regulated
YOL084W	*PHM7*	PHosphate Metabolism	*ND*	down-regulated
YBR093C	*PHO5*	PHOsphate metabolism	*MINPP1*	down-regulated
YOR161C	*PNS1*	pH Nine Sensitive	*SLC44A5*	down-regulated
YMR063W	*RIM9*	Regulator of IME2	*ND*	down-regulated
YOR312C	*RPL20B*	Ribosomal Protein of the Large subunit	*RPL18A*	up-regulated

YFL034C-A	*RPL22B*	Ribosomal Protein of the Large subunit	*RPL22; RPL22L1*	up-regulated
YOR234C	*RPL33B*	Ribosomal Protein of the Large subunit	*RPL35A*	up-regulated
YOR293W	*RPS10A*	Ribosomal Protein of the Small subunit	*RPS10; RPS10P5*	up-regulated
YJL190C	*RPS22A*	Ribosomal Protein of the Small subunit	*RPS15A*	up-regulated
YHR021C	*RPS27B*	Ribosomal Protein of the Small subunit	*RPS27; RPS27L*	up-regulated
YLR388W	*RPS29A*	Ribosomal Protein of the Small subunit	*RPS29*	up-regulated
YOR077W	*RTS2*	ND	*KIN*	down-regulated
YOR213C	*SAS5*	Something About Silencing	*MLLT1; MLLT3; YEATS2*	down-regulated
YJL089W	*SIP4*	SNF1-Interacting Protein	*ND*	down-regulated
YGR063C	*SPT4*	SuPpressor of Ty's	*SUPT4H1*	up-regulated

(ND, not determined)
